# Sandwich‐Structured Aramid Composite Papers with Heterogeneous Dielectric Layers for Integrated Corona Resistance and Thermal Conductivity

**DOI:** 10.1002/advs.77937

**Published:** 2026-10-14

**Authors:** Jun‐Wen Ren, Jun‐Xue Chen, Ying‐Ying Tong, Zi Wang, Jia‐Cheng Zhang, Lin Zhang, Bo Zhang, Fan Zhang, Guo‐Long Wang, Jun‐Wei Zha, Shen‐Li Jia

**Affiliations:** ^1^ Clean Energy Power Systems and Equipment Key Laboratory of Sichuan Province College of Electrical Engineering Sichuan University Chengdu People's Republic of China; ^2^ State Key Laboratory for Mechanical Behavior of Materials School of Materials Science and Engineering Xi'an Jiaotong University Xi'an People's Republic of China; ^3^ State Key Laboratory of Alternate Electrical Power System with Renewable Energy Sources School of Electrical and Electronic Engineering North China Electric Power University Beijing People's Republic of China

**Keywords:** aramid dielectric paper, boron nitride nanotube, corona resistance, fluorinated graphene, thermal conductivity

## Abstract

Aramid dielectric papers are indispensable for insulation in high‐performance motors for electric vehicles, aircraft, and high‐speed rail. However, the ongoing electrification trend toward higher voltage, frequency, and power density imposes severe electrothermal stresses that conventional aramid papers cannot withstand. Their inherently low thermal conductivity leads to localized heat accumulation while poor corona resistance accelerates electrothermal aging, both compromising long‐term motor reliability. Here, we report a sandwich‐structured poly(m‐phenylene isophthalamide) (PMIA) composite dielectric paper that decouples thermal conductivity from corona resistance through a functional multilayer architecture. Fluorinated graphene (FG)/PMIA surface layers provide interface passivation and charge dissipation, while boron nitride nanotube (BNNT)/PMIA core layers enable thermal conduction and dielectric reinforcement. Electrospinning followed by hot‐pressing achieves spatially oriented functional filler arrangements. FG constructs continuous charge‐transfer barrier to suppress surface discharge and BNNT constructs continuous thermal pathways in the bulk without compromising insulation integrity. The optimized dielectric paper achieves a thermal conductivity of 10.22 W·m^−^^1^·K^−^^1^ and a corona lifetime of 147.42 min, representing 1.23‐fold and 14.7‐fold improvements over neat PMIA paper, while maintaining a high breakdown strength of 199.52 kV/mm. This specific synergy originates from hierarchically oriented architecture rather than simple property addition, offering a universal strategy to design dielectric materials with decoupled multifunctionality.

## Graphical abstract

A sandwich‐structured aramid composite paper was designed to integrate high corona resistance and thermal conductivity. The fluorinated graphene/aramid surface layers effectively dissipate space charges to suppress corona discharge. Meanwhile, the boron nitride nanotube/aramid core layer constructs continuous heat‐conduction pathways. This heterogeneous multi‐layer architecture offers a promising strategy for advanced high‐power‐density motor insulation.

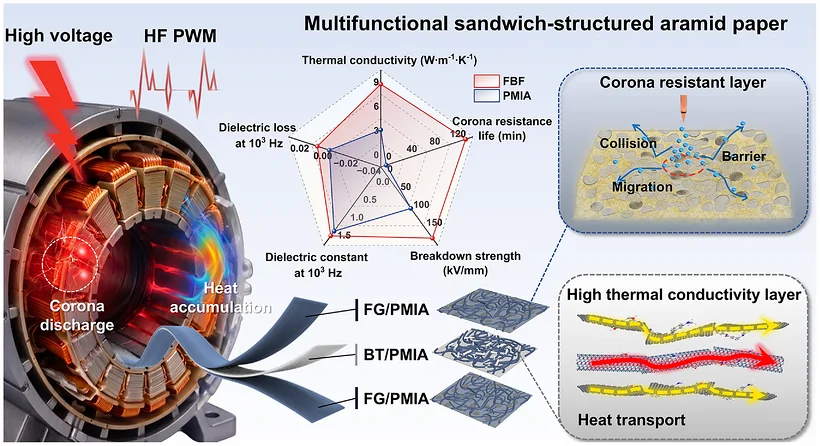

## 1 Introduction

Poly(m‐phenylene isophthalamide) (PMIA) paper is ubiquitous in the motors of electric vehicles, electric aircraft, and high‐speed trains, owing to its excellent dielectric, thermal, and mechanical properties [1, 2, 3, 4]. Yet the advancement of motors toward higher voltage and higher power density coupled with high‐frequency pulse‐width modulation imposes severe electrothermal stresses that exceed the tolerance limits of conventional PMIA paper [5, 6, 7]. High frequency and high voltage render PMIA prone to partial discharge and rapid performance deterioration while its intrinsically low thermal conductivity (λ) prevents efficient heat dissipation and accelerates electrothermal aging. These coupled deficits precipitate synergistic failure and compromise motor reliability and service life. Simultaneously achieving high corona resistance and thermal conductivity is therefore essential for next‐generation dielectric papers to ensure long‐term motor reliability [8, 9, 10, 11].

Strategies to enhance corona resistance in dielectric materials span molecular structure design, filler functionalization, and surface modification, yet the most effective approaches remain confined to functional filler incorporation [12, 13, 14, 15]. Fluorinated graphene (FG) distinguishes itself through a unique combination of two‐dimensional (2D) sheet structure, high structural stability, and extremely high C─F bond energy. This synergy regulates charge transport behavior, prolongs electrical tree growth and damage pathways in dielectric materials, beyond the capability of conventional fillers such as Al_2_O_3_, SiO_2_, and mica. Incorporating FG as a dielectric barrier achieves breakthrough enhancement in corona resistance by mitigating high‐energy particle erosion and suppressing surface partial discharges. Yet existing studies have focused primarily on FG‐mediated improvements in volume or surface resistivity and an effective description of the relationship between FG addition and corona resistance remains elusive [16, 17]. Moreover, the fluorination‐induced transition from *sp* ^2^ to *sp* ^3^ hybridization provokes intense phonon scattering and consequently impedes macroscopic λ enhancement [18, 19, 20]. Therefore, relying solely on FG protection proves insufficient for resolving localized heat accumulation under severe electrothermal conditions.

Achieving synergistic optimization of electrical erosion resistance and efficient heat dissipation necessitates a second functional component with both high intrinsic λ and electrical insulation. 1D ceramic fillers with high aspect ratios provide expansive longitudinal channels for phonon transport and reduce interfacial phonon scattering [21, 22, 23, 24, 25]. Boron nitride nanotubes (BNNT) distinguish themselves through a rare combination of exceptional λ (350 W∙m^−1^∙K^−1^), a wide bandgap of approximately 5.5 eV, and a low dielectric constant. These attributes enable simultaneous electrical insulation and heat dissipation in dielectric materials beyond the capability of other ceramic fillers. Studies demonstrate that BNNT significantly improves electrical breakdown strength and λ in dielectric materials [26, 27]. This dual functionality positions BNNT modified dielectrics as promising candidates for next‐generation highly thermally conductive systems.

Due to the fundamental differences in the transport mechanisms of charges and phonons within dielectric materials, it is greatly challenging for a single composite paper to simultaneously optimize both corona resistance and λ. Specifically, conventional strategies often rely on high filler loadings to construct phonon conduction networks for thermal enhancement. However, this readily induces interfacial defects and local electric field distortion, which inevitably comes at the expense of dielectric strength [28, 29]. In contrast, excellent corona endurance is more contingent upon preserving the high structural integrity of the dielectrics, which enables the effective inhibition of partial discharges and the retarding of surface erosion processes [30, 31]. The layered structural design has emerged precisely to overcome this thermo‐electrical trade‐off [32]. The core strategy is to spatially separate the thermally conductive layer from the charge‐blocking layer, allowing each layer to independently take on heat conduction and corona resistance, thereby avoiding performance trade‐offs inherent in single‐component materials. For example, Huang et al. [33] constructed nanofibrillated cellulose (NFC) sandwich‐structured insulation paper within a softwood pulp matrix via interlayer composite hot‐pressing. With a total NFC loading of only 10 wt%, the N/P/N structure achieved a tensile strength of 113 MPa, a DC breakdown strength of 195 kV/mm, and superior space charge suppression effects, while simultaneously reducing the relative permittivity and maintaining low dielectric loss. Although hierarchical multilayer structural design—such as sandwich architectures—has proven to be an effective paradigm for tailoring the comprehensive performance of insulating composites, an inherent physical trade‐off remains inevitable when attempting to simultaneously optimize thermal conductivity and corona resistance. Conventional sandwich architectures typically achieve merely a primitive spatial superposition of constituent phases, failing to resolve the physical trade‐off between phonon scattering and charge transport mechanisms at the microscopic scale. Furthermore, the rough interface between the thermally conductive and charge‐blocking layers often acts as a synergistic failure origin for electric field concentration and thermal resistance buildup, thereby rendering simultaneous thermal enhancement and corona resistance inherently elusive [13, 34, 35].

By decoupling microstructural functionalization—separating high‐energy surface charge dissipation from bulk thermal transport—this study breaks the traditional trade‐off between corona resistance and high thermal conductivity. Specifically, FG is enriched within the surface layer to suppress surface discharge and electrical tree injection by virtue of its deep energy traps and physical barrier effect. Concurrently, BNNT are preferentially localized in the core layer to construct continuous phonon pathways for highly efficient in‐plane heat dissipation without compromising the charge‐shielding capability of the surface layer. This spatial decoupling strategy enables the synergistic coexistence of surface corona resistance, core thermal conductivity, and overall electrical insulation, rather than forcing a trade‐off among them. Concurrently, by combining electrospinning with hot‐pressing densification, the process induces molecular chain rearrangement and enables molecular‐level fusion of PMIA across adjacent layers, thereby further enhancing interfacial adhesion and material density. Consequently, high‐performance FBF sandwich‐structured composite papers were fabricated, achieving a simultaneous enhancement in dielectric strength, corona lifetime, and thermal conductivity. Distinct from traditional high‐filler blending approaches, this study achieves site‐specific functional gains at a low total filler loading and elucidates a hierarchical synergistic mechanism of “surface barrier–core phonon pathway–interlayer molecular fusion.” Consequently, it provides a solution combining both theoretical originality and processing feasibility for next‐generation electrical insulation materials in high‐power‐density motors.

## 2 Results and Discussion

In order to achieve the synergistic enhancement of the corona resistance and thermal conductivity of the insulating paper, this paper integrates the high insulation outer layers and the high thermal conductivity intermediate layer through electrospinning and hot‐pressing densification processes, successfully preparing a FBF sandwich‐structured composite paper, and the overall preparation route is shown in Figure 1a. Introducing FG to the outer layers can significantly enhance the corona resistance of the FBF sandwich‐structured composite papers by reducing the erosion of high‐energy particles and suppressing local surface discharges. The morphologies of the different samples were observed by SEM. First, as illustrated in Figure 1b, FG exhibits a typical 2D crumpled sheet structure. This unique high‐aspect‐ratio geometric shape facilitates the formation of a dense physical barrier within the matrix, thereby effectively scattering and intercepting the bombardment of high‐energy particles. The FTIR spectra demonstrate that FG exhibits a typical high‐energy C‐F stretching vibration peak at 1207 cm^−^^1^ (Figure S3), suggesting that FG can further suppress the initiation and expansion of surface electric tree branches through deep trap energy levels and energy dissipation mechanisms, thereby delaying insulation aging. In addition, to meet the thermal management requirements within the composite paper, BNNT with high intrinsic thermal conductivity and wide bandgap were incorporated into the core layer. As illustrated in Figure 1c, BNNT exhibits a 1D high aspect ratio morphology. This structural advantage can significantly reduce the thermal percolation threshold, promote rapid interweaving of BNNT, and form a continuous and effective in‐plane phonon conduction network in the matrix under low filler content. The corresponding FTIR spectra indicate that the characteristic peaks located at 1340 and 775 cm^−^^1^ correspond to the in‐plane B–N stretching and out‐of‐plane B–N–B bending vibrations, respectively (Figure S4a), reflecting the integrity of their structure. This highly crystalline inorganic framework is accompanied by its wide bandgap (∼5.5 eV) characteristics, which significantly limit the transport of charge carriers and ensure excellent electrical insulation performance of the composite paper under harsh electric fields. The ultimate goal is to promote the establishment of internal thermal diffusion pathways while maintaining high electrical strength.

**FIGURE 1:**
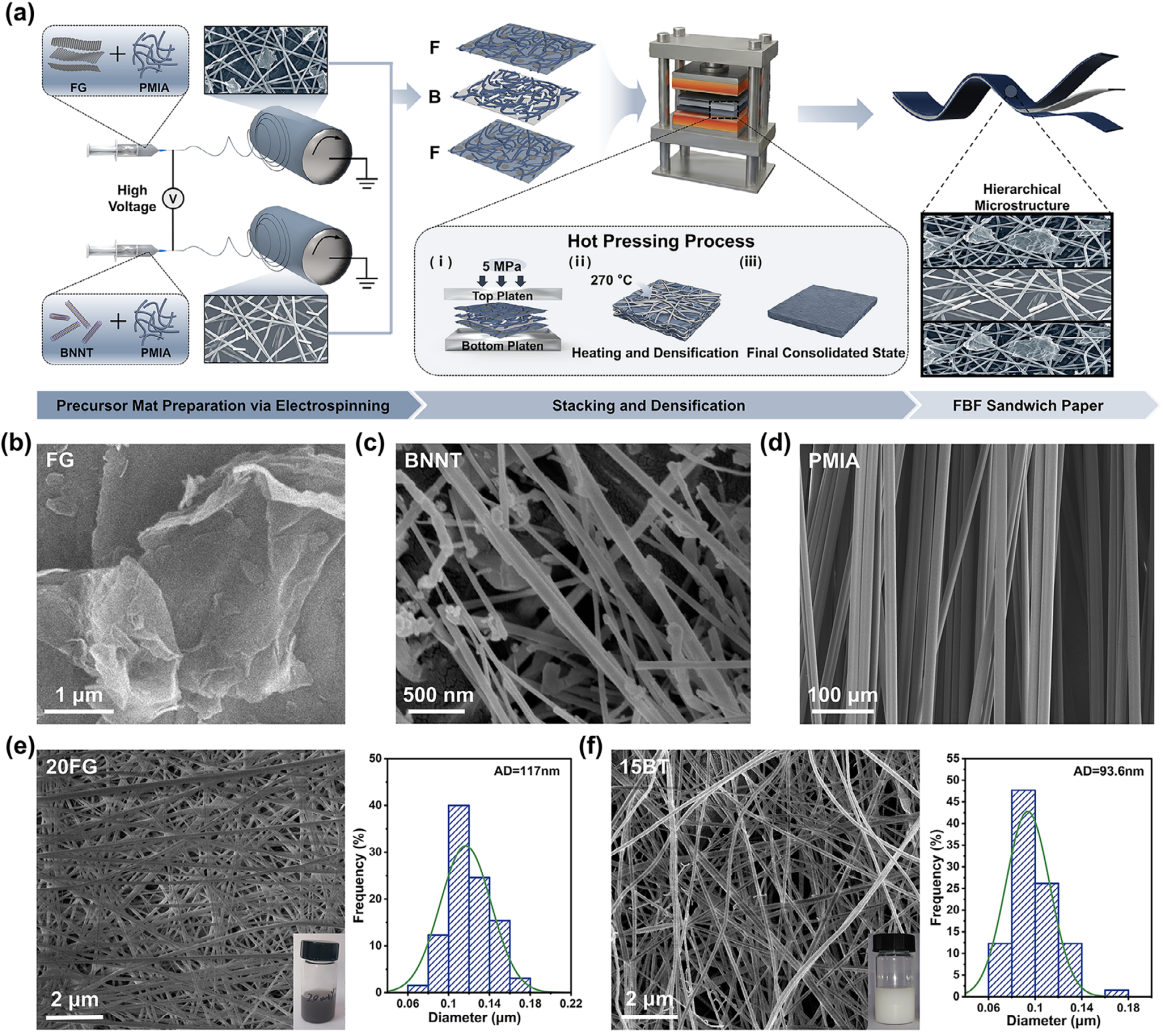
Preparation process of FBF sandwich‐structured composite paper and preliminary material characterization. (a) Schematic diagram of the preparation process of the FBF sandwich‐structured composite papers with hierarchical microstructure. SEM images of (b) FG, (c) BNNT and (d) neat PMIA electrospun fibers. SEM images and diameter statistics of (e) 20FG and (f) 15BT electrospun fibers.

Regarding the PMIA matrix, it exhibits a continuous fiber morphology and high orientation potential (Figure 1d). In this study, a finite element simulation model of the Taylor cone coupled with two‐phase flow (Figure S5) was utilized to optimize the electrospinning parameters, resulting in the successful preparation of FG/PMIA and BT/PMIA (Namely BNNT/PMIA, which will be collectively referred to as BT/PMIA in the following text) nanofiber precursor mats with uniform filler distribution. In this composite system, PVP primarily functions as a dispersant, enhancing the dispersion stability of FG and BNNT within the spinning solution through dual physical and chemical interactions. Physically, as a long‐chain polymer, PVP adsorbs onto the filler surfaces to construct a steric hindrance layer, thereby effectively preventing particle agglomeration driven by van der Waals forces. Chemically, the pyrrolidone rings in PVP molecules form π–π conjugated interactions with FG, while their carbonyl groups establish hydrogen bonds with oxygen‐containing functional groups on the filler surfaces; concurrently, the keto groups of PVP bind to BNNT surfaces via strong intermolecular forces, which substantially reinforces the interfacial affinity between the fillers and the polymer matrix [36]. As depicted in Figure S6a,b, the BNNT dispersion containing PVP maintains homogeneity upon standing, whereas the pristine counterpart exhibits pronounced sedimentation, validating the efficacy of PVP as a dispersant. Figure 1e,f displays the micromorphology and diameter statistics of 20FG and 15BT (Subsequent samples were named uniformly according to “mass fraction of filler (wt%) + filler name”, and the matrix PMIA was fixed, so it was omitted in the naming) composite fiber networks, respectively. The results reveal that the fibers possess a continuous and smooth structure without obvious bead‐on‐string or broken fiber defects. Furthermore, the diameters follow a narrow normal distribution, which strongly validates the stability and compositional uniformity of the electrospinning process. Ultimately, to integrate the loose porous nanofiber components into a dense insulating whole, a specific hot‐pressing process (270 °C, 5 MPa, 6 min) was employed for lamination densification (Figure 1a). The processing temperature was set near the glass transition temperature (T_g_) of PMIA. Under the dual drive of the pressure and temperature fields, the migration rate of polymer segments is significantly activated. This process not only eliminates the residual air in the precursor fluid, thereby eliminating the risk of air gap discharge, but also induces the transition of the graded fiber structure to a highly dense state (as shown in the illustration of the hot‐pressing process in Figure 1a). This achieves molecular level fusion between adjacent substrate layers, laying a solid structural foundation for the excellent electrical insulation and thermal conductivity of FBF sandwich‐structured composite paper (20FG surface layer and a 15BT core layer).

In order to select the FG/PMIA composite paper with the best comprehensive performance as the protective outer layer of the sandwich‐structured composite paper, FG/PMIA composite papers with different filler contents were prepared using the same process as the sandwich‐structured composite paper. The composite papers with FG contents of 10, 20, 35, and 50 wt% were named 10FG, 20FG, 35FG, and 50FG, respectively, and their various properties were subsequently evaluated. Figure 2a presents the surface SEM image of the 20FG composite paper after hot‐pressing, which demonstrates that the FG nanosheets effectively maintain their characteristic layered morphology within the composite paper. Comprehensive characterizations, including cross‐sectional SEM, FTIR, and XRD (Figure S7), indicate that the introduction of FG not only does not disrupt the structure of PMIA but also achieves deep integration between filler and the polymer matrix by enhancing the interfacial hydrogen bond interactions. Figure 2b,c presents the statistical results of corona‐resistant lifetime for the neat PMIA paper and FG/PMIA composite papers. The corona‐resistant lifetime of the neat PMIA paper is only 9.39 min, while the lifetime of the composite paper with the introduction of FG significantly increases and shows a trend of increasing first and then decreasing with increasing filler content. Among them, the 20FG composite paper reaches the highest value of 98.58 min, which is about 10.5 times that of the neat PMIA, exhibiting the optimal protective gain. The performance evolution is closely related to the charge dynamics within the material. The improved corona resistance of FG/PMIA composite papers originates from the synergistic mechanism of physical shielding and charge regulation. First, at the physical and chemical levels, FG nanosheets with high C─F bond energy can effectively resist degradation by high‐energy ions. Meanwhile, their horizontal arrangement in‐plane constructs a “brick and mortar” physical barrier (Figure 2g), which prolongs and deflects the erosion path of discharge ions through multiple collision and scattering events. Second, at the electrical level, the trap energy level structure plays a crucial role. As shown in Figure 2d and Table S1, at low filler content, the FG/PMIA composite papers interface introduces a large number of deep trap centers (with an elevated peak of energy levels, as shown in Figure S8a and Table S2). These deep traps can effectively capture charge carriers (Figure 2h) and restrict their random migration under high electric fields, thereby reducing the energy of charge carrier bombarding the matrix molecular chains. However, the lifetime decreases when the filler content exceeds 20 wt%. Combined with the carrier mobility <µ> extracted from surface potential decay (Figure 2e and Figure S8b) and the energy barrier model (Figure 2i), it can be seen that excessive fillers cause the interface area between FG sheets to overlap and reduce the energy barrier, making charge carriers migrate more easily through hopping or tunneling mechanisms (the µ value surges in the range of 35–50 wt%). Although theoretically, faster charge dissipation is beneficial for electric field homogenization, the SEM image of the 50FG paper (Figure S7b) confirms that excessive filler content causes severe filler agglomeration and micropore defects. Under high electric fields, these physical defects caused by strong electric field concentration and local discharge ultimately counteract the benefits of charge dissipation.

**FIGURE 2:**
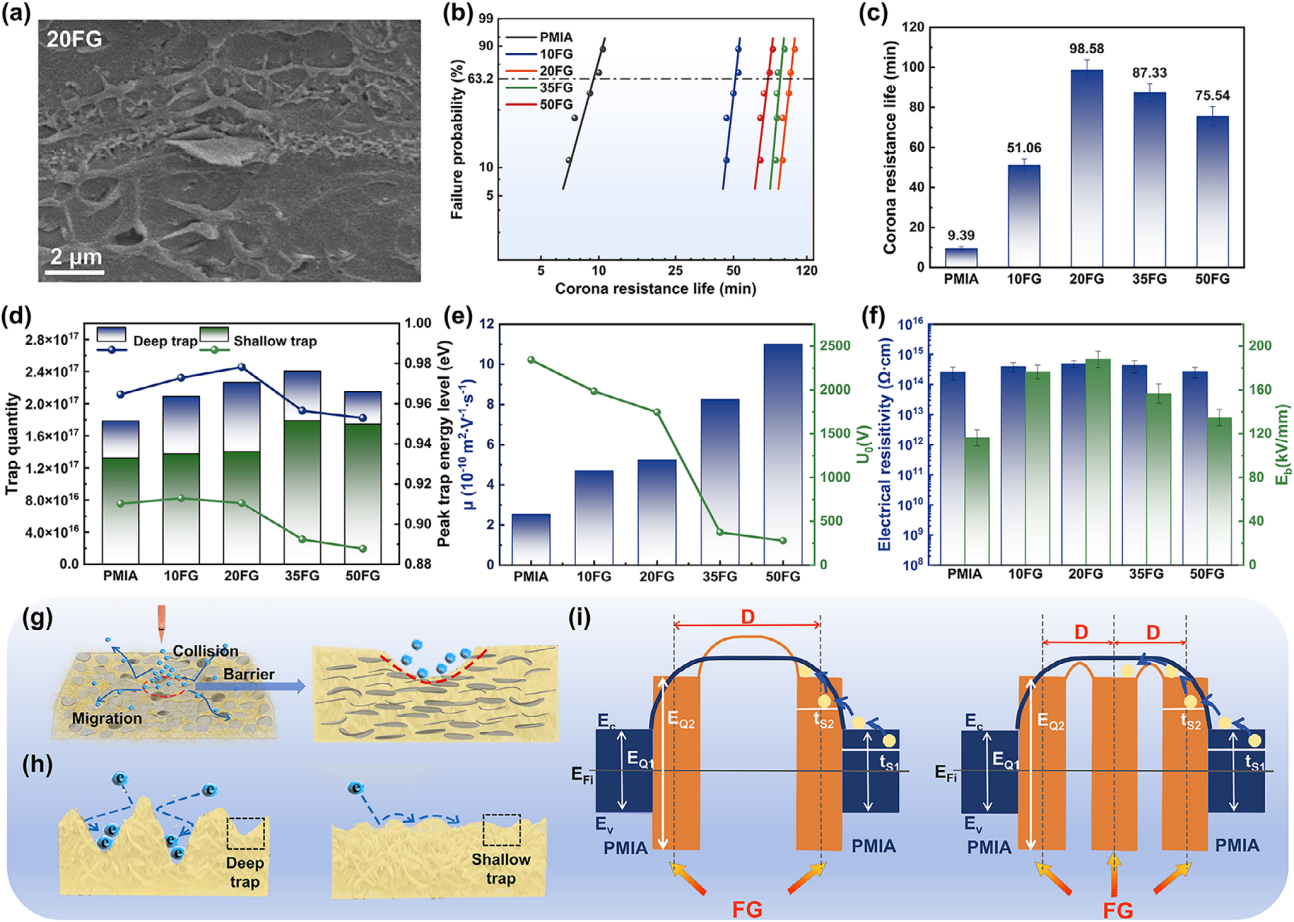
Analysis of comprehensive performance of FG/PMIA composite papers. (a) SEM image of the 20FG composite paper. (b) Failure probability of corona‐resistant lifetime deduced from Weibull distribution, (c) characteristic values of corona‐resistant lifetime, (d) trap density and peak trap energy level, (e) initial potential and carrier mobility and (f) the breakdown field strength characteristic values and volume resistivity of pure PMIA paper and the composite papers with different FG contents. (g) Corona erosion resistance mechanism and (h) carrier transport mechanism of composite paper. (i) Energy barrier diagrams of composite papers with different FG contents.

Furthermore, Figure 2f presents the dielectric breakdown strength and volume resistivity of the PMIA and various FG/PMIA composite papers. As the FG content increases, the breakdown characteristics of the FG papers exhibit a trend of increasing first and then decreasing. Quantitative analysis of trap parameters and charge dynamics reveals that the incorporation of FG reshapes the trap depth distribution and charge dissipation behavior within the composite paper. This correlates closely in a quantitative manner with its enhanced corona lifetime and dielectric breakdown strength. As shown in Table S1, pure PMIA exhibits a deep trap density of only 4.6×10^16^ m^−3^ (corresponding to a central deep trap level of 0.9646 eV). With the FG loading increasing to 20 wt%, the deep trap density sharply rises to 8.63×10^16^ m^−3^, while the central deep trap level synchronously surges to a peak of 0.9783 eV. The dual increase in deep trap density and barrier height effectively confines injected charges, maintaining the apparent carrier mobility at a low level (5.21×10^−10^ m^2^∙V^−1^∙s^−1^ for 20FG, Figure 2e). This significantly suppresses the collisional bombardment of polymer chainsby high‐energy charges under high electric fields. Consequently, the corona resistance life of 20FG dramatically increases from 9.39 min (pure paper) to 98.58 min, while its breakdown strength simultaneously reaches a peak of 187.89 kV/mm, 0.62‐fold enhancement compared to pure paper (116.27 kV/mm, Figure 2f). Conversely, when the filler loading exceeds 50 wt%, the deep trap density drops significantly to 4.03×10^16^ m^−3^, whereas the shallow trap density surges to 1.75×10^17^ m^−3^. The central energy levels for deep and shallow traps fall back to 0.9529 and 0.8878 eV, respectively, accompanied by a 0.74 eV decrease in the equivalent trap level E_trap_ (Table S2). The reduced trap barrier triggers a transition in the conduction mechanism toward hopping and tunneling transport (Figure 2i), causing a burst in the carrier mobility of 50FG to 11.02×10^−10^ m^2^∙V^−1^∙s^−1^, which corresponds to the rapid surface potential decay in Figure S8b. However, although rapid charge dissipation theoretically helps homogenize the electric field, the severe filler agglomeration and microvoid defects at high loadings (Figure S7b) dominate by inducingstrong localized electric field concentration and partial discharges. This ultimately offsets the benefit of charge dissipation, causing the corona resistance life and breakdown strength of 50FG to fall sharply back to 75.54 min and 125.75 kV/mm, respectively. The volume resistivity of the FG/PMIA composite papers is generally higher than that of the PMIA paper, indicating that the introduction of FG enhances the insulation performance. However, as the FG content continues to rise, the volume resistivity shows a decreasing trend, further suggesting that filler agglomeration and structural defects at high content levels may undermine the insulation performance. Nevertheless, the volume resistivity of all composite papers remains in the range of 10^14^ Ω·cm, which is approximately five orders of magnitude higher than that of common insulating materials (around 10^9^ Ω·cm).

In summary, the dielectric, insulation, and thermal conductivity of the FG/PMIA composite papers were systematically evaluated (Figure S9), prompting the selection of 20FG paper as the ideal functional protective surface layer for the sandwich‐structured composite paper. This choice is primarily attributed to its optimized nano‐shielding effect and trap distribution, which facilitate efficient charge regulation while precluding structural defects and maintaining favorable dielectric and insulation performance.

To meet the rapid heat dissipation requirements for the insulation of modern high‐power motors, this work introduces a BT/PMIA composite paper containing high thermal conductivity BNNT as the middle layer to construct efficient heat diffusion pathways. Similarly, BT/PMIA electrospun membranes with various filler loadings were hot‐pressed into densified composite papers using the same process. The BT/PMIA composite papers with BNNT contents of 5, 10, 15, and 20 wt% were respectively named as 5BT, 10BT, 15BT and 20BT. As evidenced by the SEM, FTIR, and XRD characterizations in Figure S10, BNNT are successfully and uniformly incorporated into the PMIA matrix, significantly enhancing the structural stability of the composite papers through interfacial hydrogen bond interactions. Figure 3a presents the in‐plane thermal conductivity (λ_||_) and the corresponding enhancement rates of the BT/PMIA composite papers with various BNNT contents. The thermal conductivity of the neat PMIA paper obtained through electrospinning combined with hot‐pressing reached 4.58 W·m^−^^1^·K^−^^1^. This is because the PMIA membrane fabricated by electrospinning is a porous network structure composed of randomly connected fibers. The thermal conductivity of air is extremely low (approximately 0.026 W·m^−^^1^·K^−^^1^), and these pores act as significant obstacles for phonon transmission. In this study, the hot‐pressing process at a temperature near the T_g_ induces intense molecular‐level interface fusion within the PMIA macromolecular chains, eliminating microscopic voids and triggering critical microstructural reconfiguration. Under the drive of the applied thermal mechanical force, the initially disordered polymer chains can rearrange. This structural transformation of the polymer chains into a more extended and oriented state minimizes phonon scattering along the chain backbone, establishing a highly efficient and continuous heat conduction pathway, thereby fundamentally enhancing the intrinsic thermal conductivity of the PMIA paper [37]. As the BNNT content increases, the thermal conductivity of the composite papers exhibits a trend of first increasing and then decreasing. At a filler content of 15 wt%, it reaches a peak value of approximately 12.2 W·m^−^^1^·K^−^^1^, which is about 160% higher than that of the neat PMIA paper. The experimental data were fitted using the Foygel model in Figure 3b, indicating that the composite papers exhibit distinct percolation behavior at low filler contents. However, the observed decline in λ_||_ at a loading of 20 wt% can be attributed to the agglomeration of high content fillers and the consequent increase in interfacial thermal resistance. The enhancement in thermal transport performance of the BT/PMIA composite paper stems from the microstructural evolution induced by the hot‐pressing process. This processing forces one‐dimensional BNNT with high aspect ratios to preferentially align along the in‐plane direction, thereby constructing a continuous and anisotropic phonon‐transport skeleton within the matrix (Figure 3c). Within the BT/PMIA layer, phonons propagate predominantly along the axial direction, featuring ultrahigh group velocities and extended mean free paths, thereby forming highly efficient, low‐thermal‐resistance pathways. Concurrently, this in‐plane orientation markedly reduces the tortuosity of phonon propagation through the polymer matrix, leading to a substantial boost in the overall heat flux density. However, phonon transport efficiency extends beyond the mere connectivity of the thermal network. On the one hand, the interfacial thermal resistance between the filler and matrix causes phonons to be partially reflected back into the original medium when crossing the interface. On the other hand, phonon scattering not only changes their propagation direction but also breaks quasi‐momentum conservation, directly shortening the effective phonon mean free path. In short, interfacial thermal resistance mainly limits the efficiency of cross‐interface transport, whereas phonon scattering dominates the loss during intra‐medium transport [38, 39]. Their combined effect creates a bottleneck in improving macroscopic thermal conductivity. To address these constraints, the BT/PMIA composite paper leverages the high aspect ratio of BNNT and their high in‐plane orientation from hot‐pressing densification. This allows heat flow to conduct long distances along the interior of BNNT, requiring phonons to cross only a few interfaces at the start and end of their paths. As a result, the probability of encountering interfacial thermal resistance and phonon scattering along the unit heat path is significantly reduced. Driven by the synergy of these advantages, the BT/PMIA composite paper achieves a dramatic boost in thermal conductivity even at a low filler loading.

**FIGURE 3:**
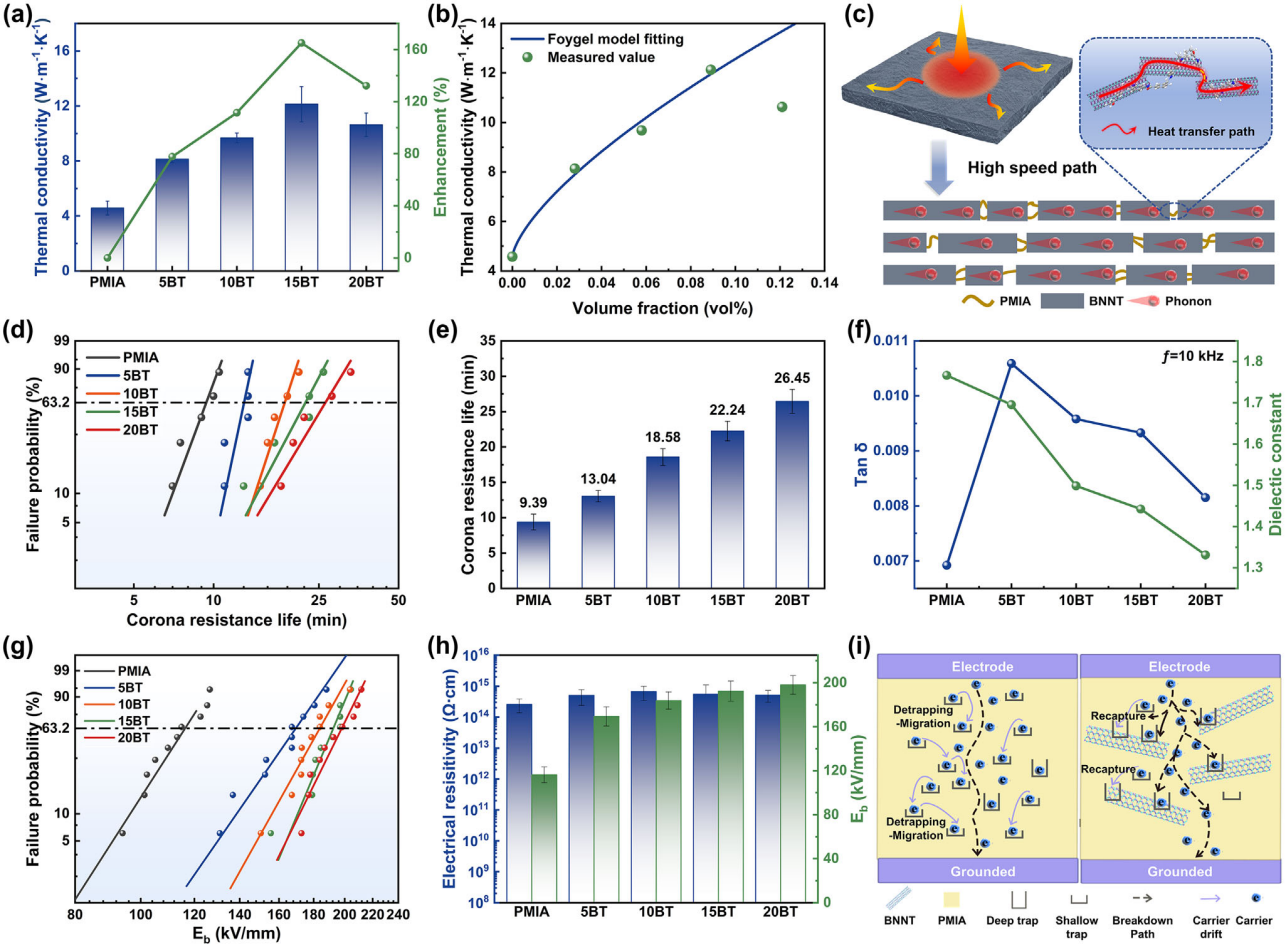
Analysis of comprehensive performance of BT/PMIA composite papers. (a) Thermal conductivity and its enhancement of PMIA paper and BT/PMIA composite papers, and (b) fitting by the Foygel model. (c) Thermal conductivity mechanism diagram of BT/PMIA composite papers. (d) Failure probability of corona‐resistant lifetime deduced from Weibull distribution and (e) characteristic values of corona‐resistant lifetime for PMIA paper and BT/PMIA composite papers. (f) Dielectric constant and dielectric loss tangent of PMIA paper and BT/PMIA composite papers at 10 kHz. (g) Failure probability of dielectric breakdown deduced from Weibull distribution. (h) The breakdown field strength characteristic values and volume resistivity of PMIA paper and BT/PMIA composite papers. (i) The insulation protection mechanism diagram of the BT/PMIA composite papers.

Furthermore, as the core layer of the insulation system, it must also maintain high electrical reliability. In this study, the corona resistance lifetime of the BT/PMIA composite papers was quantitatively evaluated using the Weibull distribution (Figure 3d,e). With the increase of BNNT content, the BT/PMIA composite papers exhibit a monotonic enhancement in their corona‐resistant endurance. The 20BT paper achieves a lifetime of 26.53 min, which is 1.83 times higher than that of the neat PMIA paper. Although the characteristic lifetime of the BT/PMIA composite papers (e.g., 22.24 min of 15BT) is lower than that of the FG/PMIA composite papers, it still exhibits a significant improvement compared to neat PMIA paper. Meanwhile, with increasing content, the dielectric loss first increases and then decreases, while remaining at a relatively low value overall. Regarding electrical breakdown characteristics, Figure 3g,h presents the breakdown strength and volume resistivity of PMIA and various BT/PMIA composite papers. As the BNNT content increases, the breakdown performance of the BT/PMIA composite papers continuously improves. Specifically, the 20BT composite paper achieves the highest breakdown strength of 197.91 kV/mm, which is 1.70 times that of the neat PMIA paper. This is because the vast interfacial area formed between the high‐aspect‐ratio BNNT and the PMIA matrix in the core layer introduces a high density of deep trap centers (Figure 3i). Under high electric fields, the charge carriers penetrating the surface layer are recaptured by these deep traps, which significantly restricts carrier mobility and reduces their average kinetic energy for impacting molecular chains. The proposed mechanism is verified by the trap energy level analysis derived from the ISPD tests (Figure S11a) and the trap parameter fitting results (Figure S11b,c and Table S3). Furthermore, the high‐insulation‐strength BNNT can force the breakdown path to transition from a unidirectional vertical development to branched and tortuous paths around the fillers. To elucidate the dynamic evolution of the breakdown path at the mesoscale, finite element analysis was employed to simulate electrical tree growth within the paper. Since BNNT have a hollow tubular structure and are randomly distributed horizontally inside the paper, the cross‐section of their 2D model cannot be simplified into a rectangle or a circle. Instead, it should be treated as an ellipse to reflect its real projected shape under random orientation. As illustrated in Figure S12, the electrical trees in the neat PMIA matrix exhibit a pronounced vertical trunk growth mode and rapidly construct a fully penetrating discharge channel. In contrast, upon the incorporation of BNNT, the highly insulating elliptical fillers construct a formidable physical barrier, forcing the electrical trees to undergo lateral bifurcation and tortuous propagation along the filler boundaries. This circuitous “barrier effect” substantially prolongs the growth trajectory of the electrical trees, thereby significantly delaying the formation of the ultimate breakdown channel (Figure 3i).

Moreover, the volume resistivity of the BT/PMIA composite papers is generally higher than that of the PMIA paper, indicating that the introduction of BNNT enhances the insulating properties of the PMIA matrix. The volume resistivity of all papers remains at the level of 10^14^ Ω·cm, which is significantly higher than the requirement stipulated by the IEC 60112 standard (10^9^ Ω·cm). Through a comprehensive evaluation of thermal diffusion efficiency and high‐field insulation characteristics, the 15BT composite paper was established as the ideal high‐thermal‐conductivity insulating core layer for the FBF sandwich‐structured composite paper. It achieves an optimal thermal conductive percolation network while simultaneously maintaining outstanding dielectric and breakdown performance.

After a comprehensive analysis of each functional monolayer paper, Figure 4a,b employs radar plots to comprehensively summarize and evaluate the dielectric, insulation, and thermal properties of each functional layer. The results demonstrate that the 20FG composite paper achieves the optimal balance between corona resistance lifetime and dielectric performance, whereas the 15BT composite paper maximizes thermal conductivity while maintaining superior breakdown strength. Consequently, the “20FG/15BT/20FG” configuration was ultimately established as the optimal component layer structure for the FBF sandwich‐structured composite papers. As displayed in Figure 4c, the FBF sandwich‐structured composite papers exhibit a well‐defined sandwich architecture with a tight interface and no obvious interlayer separation or defects. This integrated form once again confirms that the hot‐pressing process promotes the effective densification of the internal structure of the FBF sandwich‐structured composite paper. Additionally, the high‐magnification micrographs in Figure 4d demonstrate that the fillers in the hot‐pressed paper present an anisotropic arrangement within the dense substrate. This layered design is crucial for establishing efficient heat conduction channels, while maintaining excellent dielectric strength. Figure 4e,f presents the Weibull distributions and characteristic lifetime comparisons of the FBF sandwich‐structured composite paper compared with other samples. The results indicate that the characteristic lifetimes of neat PMIA, 15BT, and 20FG composite papers are 9.39 min, 22.24 min, and 98.58 min, respectively. It is notable that the FBF sandwich‐structured composite paper has a functional layered design, demonstrating the most superior corona resistance, with a characteristic lifetime as high as 147.42 min. This value not only far exceeds that of the neat PMIA matrix (approximately 15.7 times) but also significantly better than the most robust 20FG monolayer paper. The enhancement is attributed to the “defense depth” mechanism enabled by the 15BT core layer. Although the FG surface layer protective material protects against a large number of direct high‐energy particle impacts, the residual carriers leaked between the layers are effectively intercepted by the huge interface area formed between the high dielectric strength BNNT and PMIA polymer.

**FIGURE 4:**
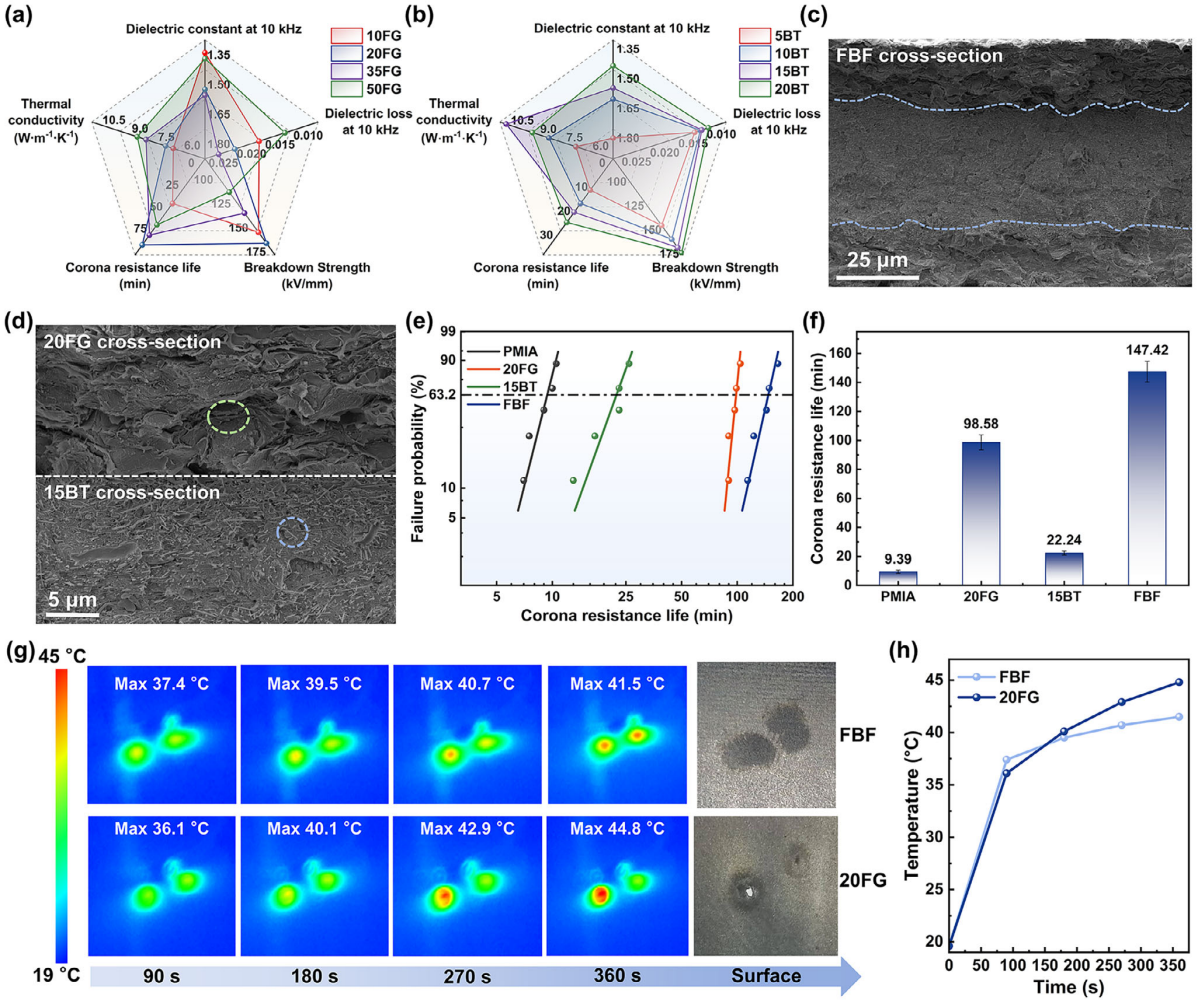
The corona resistance performance of FBF sandwich‐structured composite paper. Radar chart comparing the comprehensive performance (dielectric, thermal conductivity, corona resistance, and breakdown) of (a) FG/PMIA and (b) BT/PMIA composite papers. SEM images of cross‐sections of (c) FBF sandwich‐structured composite paper and (d) the 20FG layer and 15BT layer within them. (e) Failure probability of corona resistance life deduced from Weibull distribution and (f) characteristic values of corona resistance life of FBF sandwich‐structured composite paper and other papers. (g) Corona discharge infrared thermal imaging and (h) time‐surface temperature curves of 20FG and FBF sandwich‐structured composite papers.

As discussed previously (Figure 3i), the high‐density deep trap centers in these interface regions achieve the process of secondary recapture of high‐energy carriers. Furthermore, this multilayer structure forces the electrical tree to undergo severe deflection and branching along the insulating filler network, significantly extending the breakdown trajectory and delaying the formation of the through‐thickness failure channel. In addition to the microscopic corona resistance mechanism, owing to the hot‐pressing process, the FBF sandwich‐structured composite paper also has an extremely smooth 3D surface morphology (Figure S12a) and a very low surface roughness parameter (Figure S12d). This effectively suppresses electric field distortion at the micro‐interface, mitigates the local concentrated bombardment of high‐energy charged particles, and thereby retards the deep degradation process of the insulating material. The local accumulation of heat generated by corona discharge is a key factor leading to material aging. Infrared thermography analysis (Figure 4g) and the corresponding temperature rise curves (Figure 4h) intuitively demonstrate that the equilibrium temperature of the FBF sandwich‐structured composite paper (approximately 41.5 °C) is markedly lower than that of the 20FG monolayer paper (approximately 44.8 °C) during sustained discharge. As evidenced by the high conductivity (Figure 5a). this exceptional thermal mitigation effect stems from the continuous, low‐thermal‐resistance phonon pathways constructed by the preferentially oriented BNNT within the 15BT core layer. Rather than allowing heat to remain stagnant beneath the bombardment site, this network rapidly diffuses local thermal energy horizontally and away from the surface. As illustrated by the electro‐thermal synergistic mechanism (Figure S13), this highly efficient thermal management strategy effectively mitigates secondary damage and thermal stress induced by high‐temperature discharge, synergizing with the deep energy trap charge‐shielding effect of the surface FG to preserve overall structural integrity. Consequently, through the synergistic effect of active surface shielding, internal carrier recapture, and efficient thermal management, the FBF sandwich‐structured composite paper achieves a breakthrough enhancement in corona resistance

**FIGURE 5:**
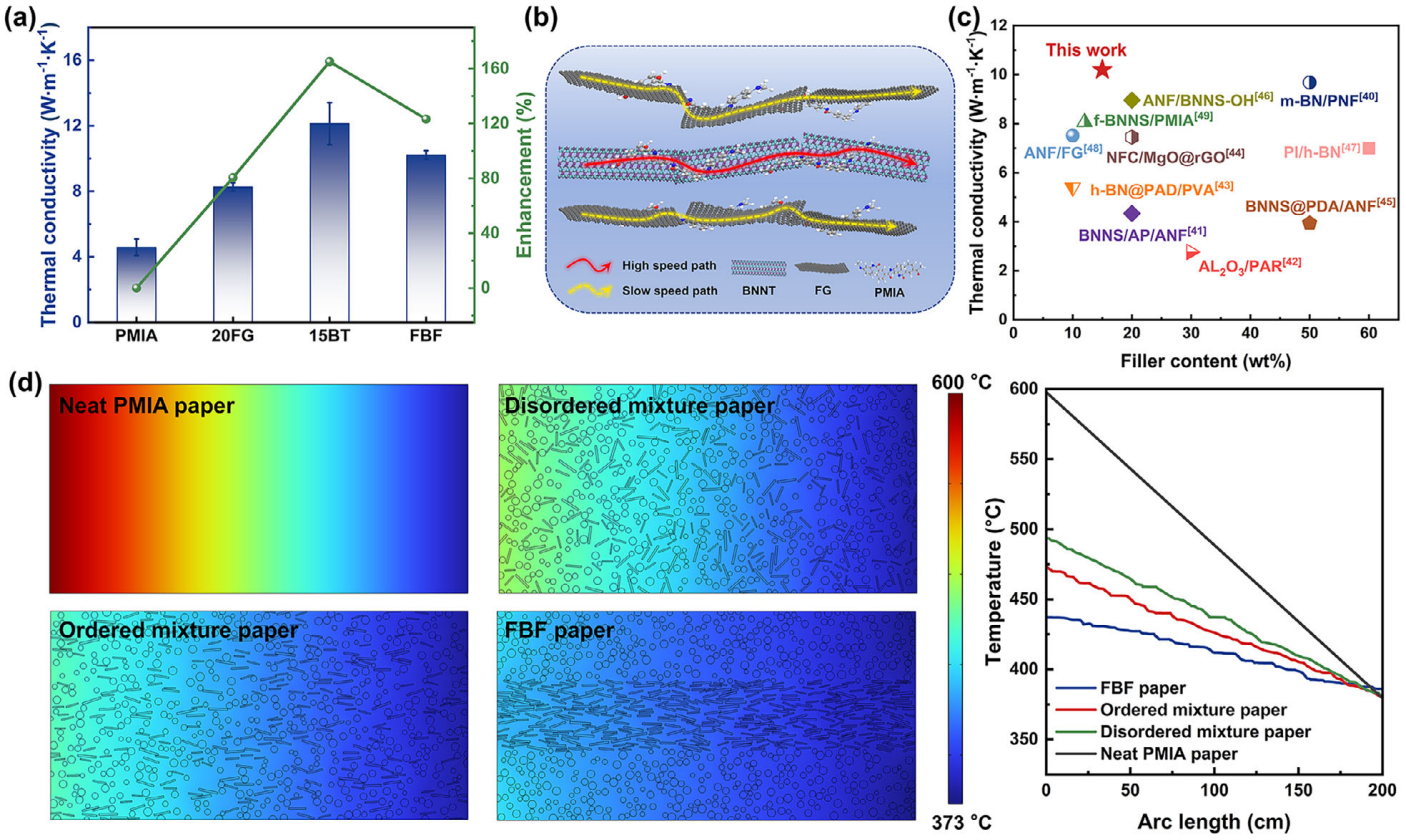
The thermal conductivity of the FBF sandwich‐structured composite paper. (a) Thermal conductivity and its enhancement of FBF sandwich‐structured composite paper and other papers. (b) Thermal conductivity mechanism diagram of FBF sandwich‐structured composite paper. (c) Comparison of the thermal conductivity of FBF sandwich‐structured composite paper with those that have been reported [40, 41, 42, 43, 44, 45, 46, 47, 48, 49]. (d) Thermal field finite element simulation of FBF sandwich‐structured composite paper.

Figure 5a compares the in‐plane thermal conductivity of neat PMIA, the 20FG, the 15BT, and the FBF sandwich‐structured composite paper. The results indicate that the FBF sandwich‐structured composite paper, consisting of 20FG surface layers and a 15BT core layer, exhibits a thermal conductivity of approximately 10.22 W·m^−^^1^·K^−^^1^. Although this value is slightly lower than that of the 15BT monolayer paper due to the presence of the low thermal conductivity surface layers, it is still significantly better than the 20FG paper and the neat PMIA paper. In conjunction with the thermal conduction mechanism (Figure 5b), although the 15BT core layer establishes an efficient phonon transport network, the macro‐thermal conductivity of the FBF sandwich‐structured composite paper is constrained from a further leap. This is primarily due to the low intrinsic thermal conductivity of the FG surface layer and the unavoidable increase in interfacial thermal resistance caused by the heterogeneous interfaces. Additionally, Figure 5c compares the thermal conductivity and filler concentration of the FBF sandwich‐structured composite paper prepared in this work with other thermally conductive composites from related literature [40, 41, 42, 43, 44, 45, 46, 47, 48, 49]. It is evident that the FBF sandwich‐structured composite paper achieves a significant enhancement in thermal conductivity at a relatively low filler content, which fully highlights the synergistic advantages of oriented filler selection and the sandwich structure design.

To further verify the superiority of the sandwich structure in thermal management from a theoretical perspective, steady‐state thermal conduction simulations were performed via Finite Element Analysis (FEA) on neat PMIA papers, direct blending models (disordered/ordered mixtures), and FBF sandwich‐structured composite papers (Figure S16). As illustrated in Figure 5d, the steady‐state temperature profiles of the four papers vary remarkably under the same heat flux. The traditional blending configuration suffers from randomly distributed fillers, which hinders the formation of effective thermal percolation networks at low filler concentrations. In contrast, the FBF sandwich architecture—characterized by the local enrichment of oriented BNNT within the core layer—effectively constructs continuous high‐density heat flow pathways in the planar direction. This architecture not only significantly reduces the phonon scattering probability during transmission process but also minimizes the in‐plane thermal resistance. Consequently, the FBF sandwich‐structured composite paper exhibits the broadest isotherm distribution and the lowest peak temperature. Its temperature decay curve of the heat source is steepest, and the heat dissipation efficiency is the highest. The above results further substantiate that this functionally layered design, characterized by a “high insulation surface layer and high thermal conductivity core layer” enables the FBF sandwich‐structured composite paper to maintain long‐term electrical reliability (such as exceptional corona resistance) via the FG surface layers, while simultaneously possessing superior macro‐thermal diffusion capabilities. This architecture realizes a profound synergistic optimization of both thermal and insulating performance.

To evaluate the insulation reliability of the FBF sandwich‐structured composite papers under high‐frequency pulse conditions in electrical motors, a comprehensive study was conducted on its wide‐frequency and wide‐temperature dielectric properties as well as dielectric breakdown strength. As illustrated in Figure 6a,b, both the dielectric constant and dielectric loss (tanδ) decrease with increasing frequency. This phenomenon is primarily attributed to typical polarization relaxation, where internal dipoles and interfacial polarization gradually fail to keep pace with the rapidly alternating external electric field at high frequencies. Below 140 °C, the FBF sandwich‐structured composite paper exhibits exceptional dielectric stability (Figure 6c), maintaining a consistently low tanδ (< 0.05) at 10^3^ Hz. However, as the temperature rises to 180 °C, the dielectric constant and loss in the low‐frequency region increase significantly. Combined with the electric modulus (M'') analysis (Figure 6d), this indicates that the elevated temperature thermal activation releases the trapped charge carriers, causing them to de‐trap at the heterogeneous interfaces (between the FG/BNNT fillers and the matrix) and subsequently accumulate in large quantities, thereby triggering a pronounced Maxwell–Wagner–Sillars (MWS) interfacial polarization effect. Dielectric breakdown strength is regarded as a key parameter for evaluating the performance of polymer‐based insulating materials. The two‐parameter Weibull distribution function was used to analyze the electrical breakdown data of the different composite papers. As shown in Figure 6e, the breakdown field strength of the FBF sandwich‐structured composite paper reaches 199.52 kV/mm, approximately 1.72 times that of neat PMIA paper. This remarkable improvement is attributed to the synergistic protective effect of the functional layered structure: the surface FG nanosheets significantly prolong the growth path of electrical trees via the “tortuous path effect”, while the dense intermediate BT/PMIA layer acts as a robust insulating barrier, and the hot‐pressing process eliminates interlayer voids. In addition, the FBF sandwich‐structured composite paper maintains a high volume resistivity exceeding 10^14^ Ω∙cm over a wide temperature range, which indirectly confirms that the sandwich structure does not introduce additional leakage channels while enhancing thermal management, thus ensuring its long‐term insulation reliability under severe electrothermal coupling conditions. Furthermore, As illustrated in Figure S14c,d, compared with pure PMIA paper, both the tensile strength and elongation at break of the FBF composite paper are significantly enhanced. Its stress–strain curve demonstrates a higher slope (indicating an elevated initial modulus) and a larger work‐of‐fracture. Pure PMIA paper exhibits a tensile strength of 20.95 MPa and a strain at break of 6.7%. In contrast, the tensile strength of the FBF composite paper increases substantially to 32.05 MPa, while maintaining a high strain at break of 6.9%. This is attributed to the hot‐pressing temperature approaching the glass transition temperature (T_g_) of PMIA, which promotes macromolecular chain rearrangement and molecular‐level fusion. Coupled with the uniform dispersion of nanofillers and interfacial hydrogen bonding, stress concentration is effectively suppressed.

**FIGURE 6:**
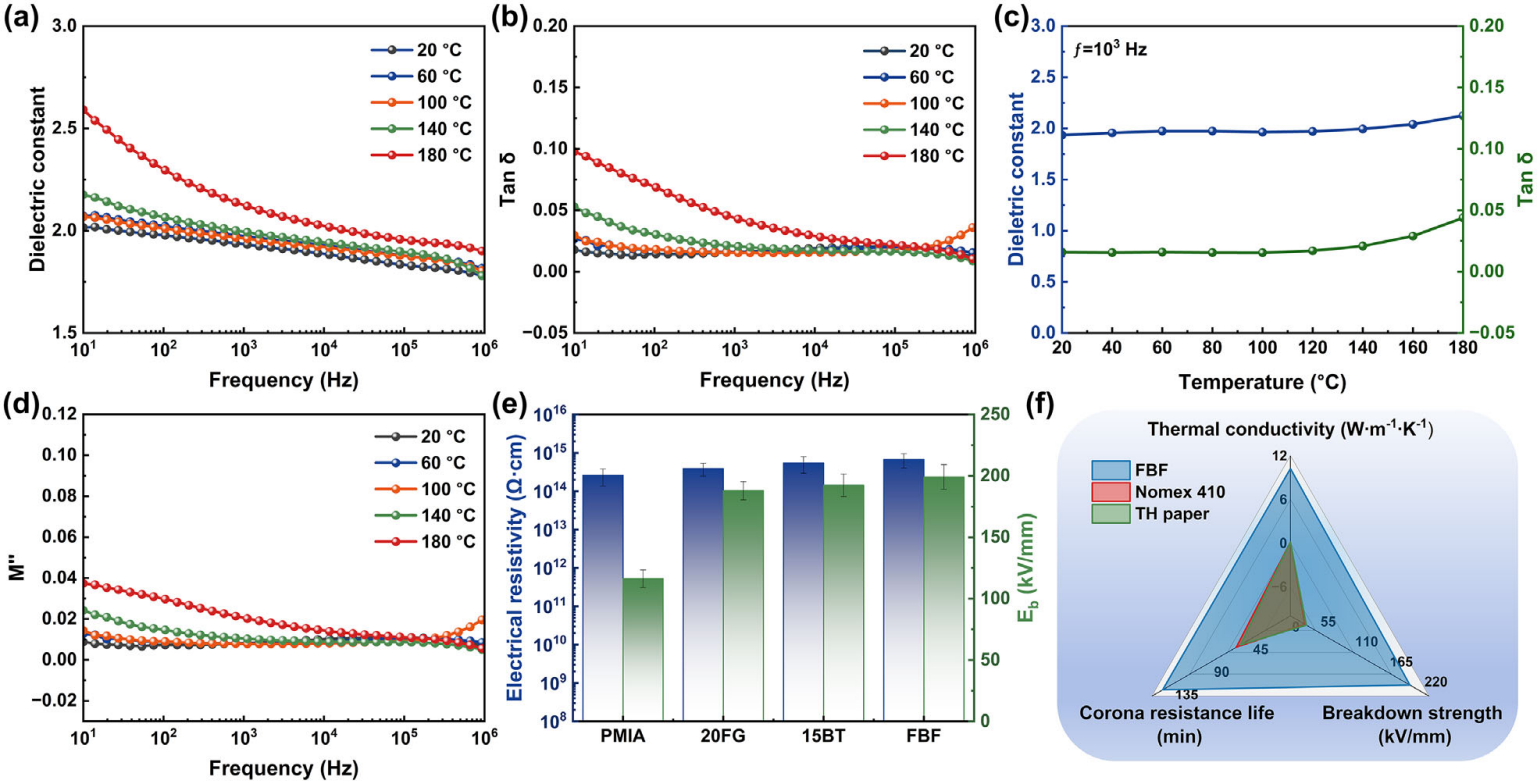
The dielectric and insulating properties of FBF sandwich‐structured composite papers. (a) Dielectric constant, (b) dielectric loss tangent of FBF sandwich‐structured composite papers at different temperatures. (c) Variation of dielectric properties of FBF sandwich‐structured composite papers with temperature at 10^3^ Hz. (d) Dielectric modulus of FBF sandwich‐structured composite papers. (e) Volume resistivity of FBF sandwich‐structured composite papers and other papers. (f) Radar chart comparing thermal and electrical properties of FBF sandwich‐structured composite paper and commercial insulating paper.

Based on the above analysis, a comprehensive performance comparison was conducted between the FBF composite paper prepared in this work and previously reported composite insulating papers (Table S4), further proving the superiority of the FBF composite paper. Furthermore, the thermal and electrical properties of the FBF sandwich‐structured composite paper are compared with typical commercial benchmarks, Nomex 410 and TH paper (aramid paper from Zhuzhou Times Huaxian Material Technology Co., Ltd.), as illustrated in Figure 6f. The results demonstrate that the FBF sandwich‐structured composite paper significantly outperforms mainstream commercially available meta‐aramid papers in key performance metrics, including thermal conductivity, corona resistance life, and breakdown strength. Additionally, we characterized the surface roughness of the FBF sandwich‐structured composite paper and two commercial papers. The 3D surface topography images (Figure S15a–c) clearly demonstrate that the FBF sandwich‐structured composite papers possess a more compact and smoother surface structure. In contrast, TH paper exhibits the poorest smoothness, which correlates with its shortest corona resistance lifetime. As shown in Figure S15d, the exceptionally low *Sa* and *Sz* values of the FBF sandwich‐structured composite paper further confirm that its excellent corona resistance indeed results from this smooth structure.

This effectively validates the unique advantages of the sandwich structural design in enhancing the comprehensive properties of materials, providing both a robust theoretical foundation and a practical paradigm for the development of next‐generation high‐performance composite insulators.

## 3 Conclusions

In conclusion, by integrating electrospinning with a hot‐pressing densification process, combined with the functional layered design of a “high insulation surface layer and high thermal conductivity core layer”, we successfully developed a multifunctional FBF sandwich‐structured composite paper. In terms of thermal conductivity, the introduction of BNNT in the core layer effectively constructs high‐density in‐plane thermal conduction pathways, significantly improving thermal diffusion capability. Finite element simulation results confirm that this structure can rapidly dissipate local heat, resulting in a high thermal conductivity of 10.22 W·m^−^^1^·K^−^^1^ for the composite paper. In terms of insulation and corona resistance, the surface FG/PMIA layer constructs a “brick and mortar” physical barrier, which effectively weakens the erosion of high‐energy particles and disperses surface discharge energy. Meanwhile, the core layer of BT/PMIA further impedes the vertical transport of carriers and delays the propagation of damage channels toward the interior. Benefiting from the synergistic effect of this surface‐core layered structure, the FBF composite paper exhibits outstanding corona resistance (a characteristic lifetime of 147.42 min), low dielectric loss over a wide temperature range (tanδ < 0.05 below 140 °C), and an extremely high volume resistivity (>10^14^ Ω·cm). Furthermore, the functional layered design significantly prolongs the growth path of electrical trees, elevating the breakdown strength to an excellent value of 199.52 kV/mm. This study provides important theoretical and experimental foundations for the synergistic “thermal conductivity‐insulation” design and application of novel slot insulation materials for high‐power motors under severe electrothermal coupling conditions.

## 4 Experimental Section

### 4.1 Materials

PMIA was supplied by Dongguan SOVETL Special Tape Co., Ltd. Few‐layer FG (F:C fluorine‐to‐carbon ratio = 1:1) was supplied by Onstar New Carbon Materials Changzhou Co., Ltd.

BNNT (BT, diameter 30–50 nm, length > 10 µm) were provided by Jiangsu XFNANO Materials Co., Ltd. N,N‐Dimethylacetamide (DMAc), m‐phenylenediamine (MPD), isophthaloyl dichloride (IPC), acetone, anhydrous lithium chloride (LiCl) and sodium hydroxide (NaOH) powder were purchased from Chengdu Cologne Chemical Reagent Co., Ltd, China. Polyvinylpyrrolidone (PVP, K30, *M*_w_ = 58000 g/mol) was obtained from Shanghai Aladdin Biochemical Technology Co., Ltd.

### 4.2 Preparation of PMIA dispersion

An appropriate amount of MPD was dissolved in DMAc under nitrogen atmosphere, and the resulting mixture solution was then cooled to 5 °C. Subsequently, IPC was slowly added dropwise under continuous magnetic stirring, followed by stirring for 1 h. NaOH powder was introduced to neutralize the mixture, and the mixture was further stirred for 0.5 h to obtain the PMIA dispersion.

### 4.3 Preparation of the FG/PMIA and BT/PMIA Composite papers

The FG/PMIA composite papers were fabricated via electrospinning combined with purification, drying, and hot‐pressing processes. First, FG powder was weighed such that its mass fractions relative to PMIA were 10, 20, 35, and 50 wt%, respectively, and PVP powder with a mass fraction of 5 wt% relative to FG was added. PVP can attach to the FG surface through physical adsorption, thereby improving its dispersion stability and reducing the tendency of FG to agglomerate during dispersion. Then, DMAc (3.2 mL) and acetone (1.6 mL) were added sequentially, followed by magnetic stirring at 700 rpm for 30 min to ensure preliminary dissolution and homogeneous mixing. Subsequently, ultrasonic treatment was performed for 30 min to promote sufficient dispersion of FG. Finally, PMIA (8 mL) dispersion with a mass concentration of 0.12 g∙mL^−1^ was added, and stirring was continued for 12 h at 60 °C to volatilize acetone from the solution as much as possible, thereby obtaining the FG spinning solution with favorable viscosity and rheological properties. The spinning solution was injected into a syringe, from which air bubbles were thoroughly removed before the syringe was mounted onto the infusion device of an electrospinning machine. The electrospinning parameters were set as follows: a positive voltage of 20 kV, a negative voltage of 13 kV, a collection distance of 12 cm. A rotating drum was used as the collector with a rotation speed of 800 rpm, and an infusion rate of 0.5 mL∙h^−1^. A double‐needle configuration was adopted for continuous electrospinning for 12 h, ultimately yielding a uniform and dense fiber web. Then, the obtained fiber web was immersed in deionized water for purification treatment for 12 h, and the water was changed several times during the purification process to ensure thorough purification. The purified fiber web was clamped between release papers, with a weight placed on top of it, and dried for 6 h at 80 °C to prevent the fiber web from warping or wrinkling during the drying process and maintain its overall flatness. The dried fiber web was subjected to lamination treatment, followed by hot‐pressing under 5 MPa for 6 min at 270 °C, and finally the FG/PMIA composite paper was obtained.

The preparation process of the BT/PMIA composite papers was basically consistent with that of the FG/PMIA papers. The only difference lies in the preparation of the spinning solution: BNNT were weighed in proportions of 5, 10, 15, and 20 wt% relative to the mass of PMIA.

### 4.4 Preparation of the FBF Sandwich‐Structured Composite Papers

After the purification and drying process, the FG/PMIA and BT/PMIA composite papers were subjected to lamination treatment, where the FG/PMIA composite papers served as the upper and lower surface layers, and the BT/PMIA composite papers as the middle layer. The laminated multilayer paper was then placed in a hot press, subjected to hot‐pressing under 5 MPa for 6 min at 270 °C, and resulted in the FBF sandwich‐structured composite papers. Among them, F represents FG/PMIA composite papers, B represents BT/PMIA composite papers.

### 4.5 Characterizations

The surface and cross‐sectional morphologies of FG, BNNT, and composite dielectric papers were characterized using a Nova NanoSEM 450 scanning electron microscope (SEM) from FEI, USA. Samples for cross‐sectional observation were immersed in liquid nitrogen for 20 min and then subjected to brittle fracture to obtain relatively flat fracture surfaces. The dielectric properties of all samples were measured using an HDTS‐600 impedance analyzer (Beijing Huace Testing Instrument Co., Ltd.). The test frequency ranged from 10 Hz to 10^6^ Hz, and the temperature‐varying test was carried out from 20 to 180 °C. The breakdown performance of the samples was evaluated using a dielectric strength test system (Beijing Beiguang Jingyi Instrument Equipment Co., Ltd.), with the operation procedure complying with the IEC 243 standard. The breakdown field strength (*E*_b_) was calculated from the breakdown voltage *U* and the sample thickness *d*.

(1) $$E_{b}=\frac{U}{d}$$

To reduce randomness and characterize the statistical characteristics of breakdown behavior, a two‐parameter Weibull distribution model was employed to statistically analyze the breakdown field strength data according to equation:[ 9]

(2) $$F\left(E\right)=1-e^{-\left(\frac{E}{\alpha }\right)\beta }$$

The fitting form of Equation 2 is as follows:

(3) $$\ln\left(-\ln\left(1-F\left(E\right)\right)\right)=\beta \left(\ln E-\ln\alpha \right)$$

where *F*(*E*) is the cumulative probability of breakdown at field strength *E*, *α* represents the characteristic breakdown field strength corresponding to a breakdown probability of 63.2%, and *β* is the shape parameter reflecting the dispersion degree of the breakdown data. In addition, the volume resistivity of the samples was measured using a Huace FE‐2000 insulation diagnostic analyzer (Beijing Huace Testing Instrument Co., Ltd.) via the two‐electrode method.

The corona resistance lifetime test was carried out on a platform constructed in accordance with the IEC 60034‐18‐42 international standard [50], using a point‐to‐plane sphere‐to‐plate electrode configuration. In each test, five groups of samples were subjected to bipolar high‐frequency square‐wave voltage with an amplitude of 1.5 times the partial discharge inception voltage (PDIV) of the samples and a frequency of 10 kHz. The test platform automatically recorded the corona resistance lifetime after breakdown occurred. The obtained lifetime data were statistically analyzed using a two‐parameter Weibull distribution model to evaluate the corona resistance performance and reliability of the samples:

(4) $$F\left(t\right)=1-e^{-\left(\frac{t}{\alpha }\right)\beta }$$

where *F*(*t*) is the cumulative probability of breakdown for the samples under corona discharge exposure time *t*, the scale parameter *α* represents the characteristic lifetime, and the shape parameter (*β*) reflects the dispersion of the corona resistance lifetime.

Isothermal surface potential decay (ISPD) measurements were performed using a needle‐plate electrode configuration. The distances between the sample and the needle electrode, as well as between the sample and the electrostatic voltmeter probe, were set to 5 and 2 mm, respectively. After being polarized under 5 kV DC voltage for 10 min, the sample was rapidly transferred beneath the electrostatic voltmeter probe, and the time‐dependent decay of the surface potential was recorded in real time. The trap distribution characteristics in the dielectric can be obtained by fitting the surface potential decay curves using Equations 5 and 6 [3].

(5) $$E_{T}=k_{B}T\ln\left(vt\right)$$

(6) $$I\left(t\right)=\left(\varepsilon_{0}\varepsilon_{r}t/eT_{h}\right)\cdot dU/dt$$

where *E_T_* denotes the trap depth, *k*_B_ is the Boltzmann constant, and *T* represents the experimental temperature. *v* (4.17×10^13^ s^−1^) stands for the attempt‐to‐escape frequency of electrons. *I*(t) is the trap density, *ԑ*_0_ is the vacuum permittivity, *ԑ*_r_ is the relative permittivity of the sample, *e* (1.6×10^−19^ C) is the elementary charge, *T*_h_ (6.626×10^−34^ J∙s) is the Planck constant, and d*U/*d*t* is the decay rate of the surface potential with time.

Thermally Stimulated Depolarization Current (TSDC) measurements were conducted using a 6517B electrometer to apply the polarization voltage and record the depolarization current, while the temperature was controlled by a TSDC testing system manufactured by Beijing Huace Testing Instrument Co., Ltd. The samples were polarized at 180 °C under a DC voltage of 400 V for 30 min and then rapidly cooled to capture the frozen charges. After short‐circuiting to eliminate residual charges, the temperature was ramped up to 230°C at a heating rate of 3°C/min. The trap energy level distribution of the materials was characterized by collecting the thermally released current. The trap charge quantity *Q*_trap_ and trap energy level *E*_trap_ were calculated according to Equations 7 and 8 [51].

(7) $$Q_{trap}=\frac{60}{T_{r}}\int_{T_{1}}^{T_{0}}I\left(t\right)dt$$

(8) $$E_{trap}=\frac{2.47T_{m}^{2}k}{\Delta T}$$

where *T*_r_ is the secondary heating rate of 3°C/min adopted in the test, *T*_0_ and *T*_1_ correspond to the onset and end temperatures of the current peak, respectively, *I*(*t*) is the depolarization current, *T*_m_ is the temperature corresponding to the current peak, *k* is the Boltzmann constant, and ∆*T* is the full width at half maximum (FWHM).

Thermal stability of the samples was evaluated using a thermogravimetric analyzer (Mettler TGA2, Switzerland).The specific heat capacity (*C*_p_, J∙kg^−1^∙K^−1^) of the samples was measured using a differential scanning calorimeter (Q2000 DSC 404 F1, TA Instruments, USA) via the sapphire method. The thermal diffusivity (*α*, mm^2^∙s^−1^) was determined by a LFA 467 laser thermal conductivity analyzer (NETZSCH, Germany), and the density (*ρ*, g∙cm^−3^) of the samples was measured based on Archimedes' principle. The thermal conductivity (*λ*, W∙m^−1^∙K^−1^) is equal to the product of thermal diffusivity, specific heat capacity, and density, with the calculation formula as follows:

(9) $$\lambda =\alpha \times C_{p}\times \rho$$

### Author Contributions

**Jun‐Wen Ren**: conceptualization, investigation, funding acquisition, methodology, writing – original draft. **Jun‐Xue Chen**: data curation, investigation, methodology. **Ying‐Ying Tong**: formal analysis. **Zi Wang**: investigation, formal analysis. **Jia‐Cheng Zhang**: formal analysis. **Lin Zhang**: formal analysis. **Bo Zhang**: formal analysis. **Fan Zhang**: conceptualization, writing – review and editing. **Guo‐Long Wang**: conceptualization, writing – review and editing. **Jun‐Wei Zha**: conceptualization, writing – review and editing. **Shen‐Li Jia**: supervision, writing – review and editing.

## Funding

This work was supported by the National Key R&D Program on Smart Grid of China (2025ZD0808200), the National Natural Science Foundation of China (No. 52377028), and the Natural Science Foundation of Sichuan Province of China (2025ZNSFSC0354).

## Conflicts of Interest

The authors declare no conflicts of interest.

## Supporting information




**Supporting File 1**: advs77937‐sup‐0001‐SuppMat.docx.

**Supporting File 2**: advs77937‐sup‐0002‐SuppMat.docx.

## Data Availability

The data that support the findings of this study are available from the corresponding author upon reasonable request.

## References

[1] A. He , T. Xing , Z. Liang , et al., “Advanced Aramid Fibrous Materials: Fundamentals, Advances, and Beyond,” Advanced Fiber Materials 6, no. 1 (2024): 3–35, 10.1007/s42765-023-00332-1.

[2] B. Yang , X. Ding , M. Zhang , and L. Wang , “Scalable Electric Heating Paper Based on CNT/Aramid Fiber With Superior Mechanical and Electric Heating Properties,” Composites Part B: Engineering 224 (2021): 109242, 10.1016/j.compositesb.2021.109242.

[3] K. Sun , F. Lv , W. Zhang , et al., “Self‐Reinforced Doping Strategy in the Multiscale PMIA Paper for High Mechanical Properties and Insulating Performance,” ACS Applied Materials & Interfaces 15, no. 46 (2023): 53902–53912, 10.1021/acsami.3c11566. 37935440

[4] H.‐O. Ruan , Q. Xie , S.‐S. Wang , et al., “Experimental Study on Damage Mechanism of Partial Discharge of Oil‐Immersed Laminated PMIA Paper,” IEEE Transactions on Dielectrics and Electrical Insulation 28, no. 4 (2021): 1223–1230, 10.1109/TDEI.2021.009548.

[5] C. Chen , B. Qi , C. Gao , and C. Li , “DC Electric Field Calculation Model of Oil‐Paper Insulation Considering Charge Transport Behavior,” IEEE Transactions on Dielectrics and Electrical Insulation 32, no. 6 (2025): 3383–3392, 10.1109/TDEI.2025.3579470.

[6] Q. Wang , Z. Yang , W. Hu , Z. Xu , P. Liu , and Z. Peng , “Heat Transfer Characteristics of Novel Resin Impregnated Paper Converter Transformer Valve‐Side Bushing With Two‐Phase Closed Thermosyphon,” High Voltage 10, no. 5 (2025): 1221–1234, 10.1049/hve2.70011.

[7] H. Cui , L. Yang , Y. Zhu , S. Li , A. Abu‐Siada , and S. Islam , “A Comprehensive Analyses of Aging Characteristics of Oil‐Paper Insulation System in HVDC Converter Transformers,” IEEE Transactions on Dielectrics and Electrical Insulation 27, no. 5 (2020): 1707–1714, 10.1109/TDEI.2020.008788.

[8] B. Yang , Y. Wang , Y. Zhou , J. He , P. Xu , and M. Zhang , “Micro/Nanofiber‐Network Assembled Aramid Paper With Excellent Mechanical and Electrical Insulating Properties,” ACS Nano 19, no. 42 (2025): 37027–37039, 10.1021/acsnano.5c10576. 41100752

[9] H. Ruan , F. Lü , J. Song , et al., “Enhanced Thermal Conductance and Electrical Insulation of AlN/PMIA Composite Paper via Nano Splitting of Matrix and Size Grading of Fillers,” Composites Science and Technology 224 (2022): 109477, 10.1016/j.compscitech.2022.109477.

[10] Y. Shi , Y. Zhao , C. Gong , et al., “Effect of POSS on the Dielectric Properties of Thermally Aged Epoxy Resin Insulating Materials,” IEEE Transactions on Dielectrics and Electrical Insulation 29, no. 5 (2022): 1763–1770, 10.1109/TDEI.2022.3194492.

[11] F. Lü , R. Yang , H. Ruan , S. Wang , and X. Yu , “Effect of Anti‐Hydrolysis AlN Microspheres on the Electrical Insulation and Thermal Conductivity of PMIA Paper,” IEEE Transactions on Dielectrics and Electrical Insulation 30, no. 4 (2023): 1674–1680, 10.1109/TDEI.2023.3243194.

[12] Y. Guo , X. Zhang , H. Xu , et al., “Design of Polyimide‐Based Composite Insulating Paper with High Corona Life Resistance,” ACS Applied Polymer Materials 7, no. 16 (2025): 10868–10878, 10.1021/acsapm.5c02187.

[13] X.‐F. Pan , Z. Bao , W. Xu , et al., “Recyclable Nacre‐Like Aramid‐Mica Nanopapers with Enhanced Mechanical and Electrical Insulating Properties,” Advanced Functional Materials 33, no. 9 (2023): 2210901, 10.1002/adfm.202210901.

[14] A. Bjurström , H. Edin , H. Hillborg , et al., “A Review of Polyolefin‐Insulation Materials in High Voltage Transmission; From Electronic Structures to Final Products,” Advanced Materials 36, no. 52 (2024): 2401464, 10.1002/adma.202401464. 38870339

[15] Z. Han , Y. Niu , X. Shi , et al., “MXene@c‐MWCNT Adhesive Silica Nanofiber Membranes Enhancing Electromagnetic Interference Shielding and Thermal Insulation Performance in Extreme Environments,” Nano‐Micro Letters 16, no. 1 (2024): 195, 10.1007/s40820-024-01398-1. PMC11557810 38743205

[16] Q. Li , Y. Qi , Z. Wang , et al., “Simultaneous Enhancement of Dielectric and Thermal Conductivity in Poly(Vinylidene Fluoride) Composite Films via Fluorinated Graphene Nanosheets Incorporating,” Nano Research 18, no. 6 (2024): 94907449, 10.26599/NR.2025.94907449.

[17] B. Du , N. Chen , Y. Mai , G. Zhang , and Y. Zhao , “Improving the Hydrophobicity and Insulation Properties of Epoxy Resins by the Self‐Assembly‐Induced Coating of Fluorinated Graphene,” ACS Applied Materials & Interfaces 15, no. 27 (2023): 32895–32902, 10.1021/acsami.3c04623. 37377187

[18] M. Narasaki , H. Wang , T. Nishiyama , T. Ikuta , and K. Takahashi , “Experimental Study on Thermal Conductivity of Free‐Standing Fluorinated Single‐Layer Graphene,” Applied Physics Letters 111, no. 9 (2017): 093103, 10.1063/1.5001169.

[19] J. Huang , C. Li , J. Liu , and Y. Xie , “Fluorinated Graphene Films With High Temperature Sensitivity for Uncooled Bolometer Applications,” ACS Applied Nano Materials 7, no. 14 (2024): 16215–16225, 10.1021/acsanm.4c02145.

[20] X. Chen , K. Fan , Y. Liu , et al., “Recent Advances in Fluorinated Graphene From Synthesis to Applications: Critical Review on Functional Chemistry and Structure Engineering,” Advanced Materials 34, no. 1 (2022): 2101665, 10.1002/adma.202101665. 34658081

[21] N. Burger , A. Laachachi , M. Ferriol , M. Lutz , V. Toniazzo , and D. Ruch , “Review of Thermal Conductivity in Composites: Mechanisms, Parameters and Theory,” Progress in Polymer Science 61 (2016): 1–28, 10.1016/j.progpolymsci.2016.05.001.

[22] Z. Wang , Z. Wu , L. Weng , et al., “A Roadmap Review of Thermally Conductive Polymer Composites: Critical Factors, Progress, and Prospects,” Advanced Functional Materials 33, no. 36 (2023): 2301549, 10.1002/adfm.202301549.

[23] Z. Chen , W. Ren , L. Gao , B. Liu , S. Pei , and H.‐M. Cheng , “Three‐Dimensional Flexible and Conductive Interconnected Graphene Networks Grown by Chemical Vapour Deposition,” Nature Materials 10, no. 6 (2011): 424–428, 10.1038/nmat3001. 21478883

[24] X. Hao , S. Wan , Z. Zhao , et al., “Enhanced Thermal Conductivity of Epoxy Composites by Introducing 1D AlN Whiskers and Constructing Directionally Aligned 3D AlN Filler Skeletons,” ACS Applied Materials & Interfaces 15, no. 1 (2023): 2124–2133, 10.1021/acsami.2c18356. 36576869

[25] X. Hu , M. Huang , N. Kong , F. Han , R. Tan , and Q. Huang , “Enhancing the Electrical Insulation of Highly Thermally Conductive Carbon Fiber Powders by SiC Ceramic Coating for Efficient Thermal Interface Materials,” Composites Part B: Engineering 227 (2021): 109398, 10.1016/j.compositesb.2021.109398.

[26] J.‐W. Ren , H.‐C. Wei , S. Yang , et al., “Core‐Shell Engineered Boron Nitride Nanotubes for Enhancing Dielectric Properties of Epoxy Composites,” Materials & Design 253 (2025): 113942, 10.1016/j.matdes.2025.113942.

[27] A. Zandieh , P. Buahom , E. Baradaran Shokouhi , et al., “Highly Anisotropic Thermally Conductive Dielectric Polymer/Boron Nitride Nanotube Composites for Directional Heat Dissipation,” Small 20, no. 48 (2024): 2404189, 10.1002/smll.202404189. 39109567

[28] X. Qian , J. Zhou , and G. Chen , “Phonon‐Engineered Extreme Thermal Conductivity Materials,” Nature Materials 20, no. 9 (2021): 1188–1202, 10.1038/s41563-021-00918-3. 33686278

[29] R. Wang , Y. Zhu , S. Huang , et al., “Dielectric Polymers With Mechanical Bonds for High‐Temperature Capacitive Energy Storage,” Nature Materials 24, no. 7 (2025): 1074–1081, 10.1038/s41563-025-02130-z. 39953246

[30] Z. Li , B. Zhu , S. Wu , et al., “Enhanced DC Surface Discharge Characteristics of Epoxy Composites Treated by Dielectric Barrier Discharge Plasma Fluorination and Its Mechanisms,” Applied Surface Science 680 (2025): 161489, 10.1016/j.apsusc.2024.161489.

[31] X. Zhao , L. Zhang , Z. Fan , et al., “Excellent High‐Temperature Dielectric Energy Storage Performance in Bilayer Nanocomposites With High‐Entropy Ferroelectric Oxide Fillers,” Nature Communications 16, no. 1 (2025): 5570, 10.1038/s41467-025-60683-8. PMC12215717 40593587

[32] D. Yang , Z. Zhao , L. Zhu , et al., “Tough and Tear Resistant Hydrogel With a Sandwich Mineralized Structure Induced by Bidirectional Ion Migration,” Nature Communications 16, no. 1 (2025): 11328, 10.1038/s41467-025-66423-2. PMC12722394 41309633

[33] X. Huang , Y. Zhou , Q. Gesang , et al., “Construction of Nanocellulose Sandwich‐Structured Insulating Paper and Its Enhancement for Mechanical and Electrical Properties,” IEEE Transactions on Dielectrics and Electrical Insulation 28, no. 4 (2021): 1127–1135, 10.1109/TDEI.2021.009446.

[34] H. Ruan , S. Hu , J. Song , and X. Liu , “Impact of Defect Structures on Interfacial and Insulation Properties in Epoxy‐Impregnated Aramid Paper,” IEEE Transactions on Dielectrics and Electrical Insulation 31, no. 4 (2024): 1815–1822, 10.1109/TDEI.2024.3382218.

[35] D. Zhang , S. Wang , J. Jiang , et al., “Enhancement of Mechanical and Electrical Properties of Multilayer Composite Nanocellulose‐Modified Insulating Paper,” IEEE Transactions on Dielectrics and Electrical Insulation 33, no. 1 (2026): 257–266, 10.1109/TDEI.2025.3645748.

[36] H. Lim , Y.‐K. Kim , H.‐S. Kim , et al., “Lyotropic Boron Nitride Nanotube Liquid Crystals: Preparation, Characterization, and Wet‐Spinning for Fabrication of Composite Fiber,” ACS Applied Materials & Interfaces 15, no. 20 (2023): 24681–24692, 10.1021/acsami.3c00189. 37163756

[37] Y. Xu , D. Kraemer , B. Song , et al., “Nanostructured Polymer Films with Metal‐Like Thermal Conductivity,” Nature Communications 10, no. 1 (2019): 1771, 10.1038/s41467-019-09697-7. PMC6467866 30992436

[38] Y. Yan , Y. Chen , Z. Mao , et al., “Bridging the Thermal Divide: Nano‐Architectonics and Interface Engineering Strategies for High‐Performance 2D Material‐Based Polymer Composites,” ACS Nano 19, no. 22 (2025): 20357–20402, 10.1021/acsnano.5c02539. 40411496

[39] Z. Yao , B. Liu , S. Zhang , et al., “Regulating Phonon‐Carrier Transport by Interfacial Symmetry Breaking in Thermoelectric Multilayers,” Science Advances 12, no. 34 (2026): aeh5460, 10.1126/sciadv.aeh5460. PMC13496199 42627918

[40] L. Tang , K. Ruan , X. Liu , Y. Tang , Y. Zhang , and J. Gu , “Flexible and Robust Functionalized Boron Nitride/Poly(p‐Phenylene Benzobisoxazole) Nanocomposite Paper With High Thermal Conductivity and Outstanding Electrical Insulation,” Nano‐Micro Letters 16, no. 1 (2024): 38, 10.1007/s40820-023-01257-5. PMC10689708 38032407

[41] M. Li , Y. Zhu , and C. Teng , “Facial Fabrication of Aramid Composite Insulating Paper with High Strength and Good Thermal Conductivity,” Composites Communications 21 (2020): 100370, 10.1016/j.coco.2020.100370.

[42] L. Cheng , H. Ma , X. Liu , et al., “High‐Performance Al_2_O_3_/Polyarylate Nanocomposite Paper With Enhanced Insulation, Thermal Conductivity, and Environmental Stability for Advanced Electrical Applications,” Chemical Engineering Journal 511 (2025): 162000, 10.1016/j.cej.2025.162000.

[43] H. Shen , J. Guo , H. Wang , N. Zhao , and J. Xu , “Bioinspired Modification of H‐BN for High Thermal Conductive Composite Films With Aligned Structure,” ACS Applied Materials & Interfaces 7, no. 10 (2015): 5701–5708, 10.1021/am507416y. 25707681

[44] M. Ma , L. Xu , L. Qiao , et al., “Nanofibrillated Cellulose/MgO@rGO Composite Films with Highly Anisotropic Thermal Conductivity and Electrical Insulation,” Chemical Engineering Journal 392 (2020): 123714, 10.1016/j.cej.2019.123714.

[45] T. Ma , Y. Zhao , K. Ruan , et al., “Highly Thermal Conductivities, Excellent Mechanical Robustness and Flexibility, and Outstanding Thermal Stabilities of Aramid Nanofiber Composite Papers With Nacre‐Mimetic Layered Structures,” ACS Applied Materials & Interfaces 12, no. 1 (2020): 1677–1686, 10.1021/acsami.9b19844. 31820630

[46] L. Zhao , C. Wei , J. Ren , et al., “Biomimetic Nacreous Composite Films Toward Multipurpose Application Structured by Aramid Nanofibers and Edge‐Hydroxylated Boron Nitride Nanosheets,” Industrial & Engineering Chemistry Research 61, no. 25 (2022): 8881–8894, 10.1021/acs.iecr.2c01281.

[47] K. Sato , H. Horibe , T. Shirai , et al., “Thermally Conductive Composite Films of Hexagonal Boron Nitride and Polyimide With Affinity‐Enhanced Interfaces,” Journal of Materials Chemistry 20, no. 14 (2010): 2749, 10.1039/b924997d.

[48] L. Zhao , W. Wu , L. Jia , et al., “Mechanically Strong and Thermally Conductive Paper Made From Aramid Nanofiber and Fluorinated Graphene With Excellent Dielectric Properties,” Composite Interfaces 29, no. 6 (2022): 659–673, 10.1080/09276440.2021.1990482.

[49] G. Duan , Y. Wang , J. Yu , J. Zhu , and Z. Hu , “Novel Poly(m‐phenyleneisophthalamide) Dielectric Composites With Enhanced Thermal Conductivity and Breakdown Strength Utilizing Functionalized Boron Nitride Nanosheets,” Macromolecular Materials and Engineering 304, no. 11 (2019): 1900310, 10.1002/mame.201900310.

[50] X. Li , T. He , Q. Yin , et al., “Fluorinated Al_2_O_3_/Siloxane Modified PI Films towards Vastly Enhanced Corona Resistance Performance,” Composites Part A: Applied Science and Manufacturing 172 (2023): 107613, 10.1016/j.compositesa.2023.107613.

[51] X. Du , Z. Chai , J. Zhang , et al., “Synergistic Enhancements of Mechanical and Insulating Properties in Electrospun PMIA‐Based Composite Films,” Journal of Physics D: Applied Physics 58, no. 28 (2025): 285501, 10.1088/1361-6463/ade9d5.

