# Supplementary material for: Sandwich‐Structured Aramid Composite Papers with Heterogeneous Dielectric Layers for Integrated Corona Resistance and Thermal Conductivity
Source: Adv Sci (Weinh). 2026 Oct 14:e77937. Online ahead of print. doi: 10.1002/advs.77937 (PMC13662768; doi:10.1002/advs.77937)
Supplement: Supplementary file 1 [file ADVS-9999-e77937-s001.docx]

**Supporting Information**

**Sandwich-Structured Aramid Composite Papers with Heterogeneous Dielectric Layers for Integrated Corona Resistance and Thermal Conductivity**

*Jun-Wen Ren^1^, Jun-Xue Chen^1^, Ying-Ying Tong^1^, Zi Wang^1^, Jia-Cheng Zhang^1^, Lin Zhang^1^, Bo Zhang^1^, Fan Zhang^1,^*, Guo-Long Wang^2,^*, Jun-Wei Zha^3,^*, Shen-Li Jia^1,^**

*^1^Clean Energy Power Systems and Equipment Key Laboratory of Sichuan Province, College of Electrical Engineering, Sichuan University, Chengdu 610065, P.R. China.*

*^2^State Key Laboratory for Mechanical Behavior of Materials, School of Materials Science and Engineering,* *Xi’an Jiaotong University, Xi’an 710049, P.R. China.*

*^3^State Key Laboratory of Alternate Electrical Power System with Renewable Energy Sources, School of Electrical and Electronic Engineering, North China Electric Power University, Beijing 102206, P.R. China.*

**** Corresponding authors.***

E-mail: [fzhang202507@163.com](mailto:fzhang202507@163.com) (F. Zhang);

[guolongwang@xjtu.edu.cn](mailto:guolongwang@xjtu.edu.cn) (G. Wang);

zhajw@ncepu.edu.cn (J. Zha);

[jiashenli@scu.edu.cn](mailto:jiashenli@scu.edu.cn) (S. Jia).

**Figure S1.** The molecular weight distribution chart of PMIA dispersion.

As shown in **Figure S1**, the molecular weight distribution curve presents a perfect smooth single peak. This further confirms the uniformity of the polymerization process, without phase separation or local chemical stoichiometric imbalance. The curve converges sharply in the high molecular weight region, but there is a slight natural tailing in the low molecular weight region (around 10^4^). This is an inevitable statistical characteristic of the condensation reaction, indicating that a small amount of shorter molecular chains is retained in the system.

**Figure S2.** ^1^H NMR spectrum of PMIA dispersion.

The chemical structure of the poly(m-phenylene isophthalamide) (PMIA) resin was confirmed by ^1^H-NMR spectroscopy. As shown in **Figure S2**, the characteristic singlet at 10.58 ppm is assigned to the amide protons (-NH-) in the polymer backbone. Driven by the shielding and deshielding effects of the adjacent carbonyl and imino groups, the aromatic protons on the isophthaloyl and m-phenylenediamine moieties exhibit distinct multiplet signals in the regions of 8.00–8.55 ppm (downfield) and 7.21–7.73 ppm (upfield). The integration area ratio of the amide proton to the total aromatic protons is approximately 1:4, which is in excellent agreement with the theoretical repeating unit of PMIA. Additionally, the minor peaks at 2.95, 2.78, and 1.95 ppm are attributed to trace amounts of residual N,N-dimethylacetamide (DMAc) solvent trapped within the polymer network.^[1–3]^

**Figure S3. (a)** FTIR spectrum of FG. **(b)** TGA curve of FG.

The FTIR spectrum reveals distinct absorption peaks within the range of 1000–1300 cm^-1^. Specifically, the strong absorption peak at 1207 cm⁻¹ is assigned to the C–F bonds stretching vibration (**Figure S3a**).^[1]^ The TGA curve illustrates that FG undergoes a relatively slow weight loss from 100 to 500 °C. Once the temperature exceeds 550 °C, a sharp mass loss is observed, accompanied by a low residual char yield at elevated temperatures. These results indicate that FG possesses moderate thermal stability below 500 ℃, whereas the rapid weight loss at high temperature corresponds to the initiation of the thermal decomposition (**Figure S3b**). This process is typically associated with the cleavage of C–F bonds and the further thermal cracking of the carbon skeleton.

**Figure S4.** **(a)** FTIR spectrum of BNNT. **(b)** TGA curve of BNNT.

In the FTIR spectrum, the characteristic peaks at 1340 cm^-1^ and 775 cm^-1^ are assigned to the in-plane B–N stretching vibration and the out-of-plane B–N–B bending vibration, respectively, confirming the structural features of the BNNT (**Figure S4a**).^[2]^ The TGA curve reveals that BNNT undergo negligible thermal decomposition up to 800 ℃, demonstrating their exceptional thermal stability (**Figure S4b**).

**Figure S5.** Simulation results of the Taylor cone in electrospinning.

To analyze the formation process of the Taylor cone under various voltages, a simulation model based on two-phase flow coupling was established. The model incorporates key factors, including the flow behavior of electrospinning solution, electric field effect, and surface tension. The simulation results facilitate the evaluation of fiber-forming conditions and provide a theoretical reference for optimizing electrospinning parameters.^[3]^ **Figure S5** illustrates the morphological evolution of the solution interface at the needle tip under different applied voltages. At low voltages, surface tension dominates the fluid interface, forming a blunt and rounded meniscus that inhibits the formation of a Taylor cone. Under an optimal voltage, a stable Taylor cone is gradually established. However, excessively high voltage induces over-stretching of the spinning solution, which sharpens the Taylor cone and further results in unstable and discontinuous electrospinning. Combined with the morphological evolution of the Taylor cone and the requirement for stable electrospinning, the optimal processing parameters were determined as follows: an applied voltage of 20 kV, ambient temperature of 35 ℃, needle-to-collector distance of 12 cm, and solution flow rate of 0.5 mL/h. A rotating drum collector was utilized with a rotational speed of 800 rpm. To enhance spinning efficiency, a dual-needle configuration was employed for 12 h of continuous spinning, and ultimately the electrospun fiber mat was obtained.

**Figure S6.** **(a)** The FG/PMIA electrospun solutions with different FG contents. **(b)** Comparison of 15BT electrospun solutions with PVP (left) and without PVP (right). **(c)** The Tyndall effect of PMIA dispersion liquid. SEM images of **(d)** different FG/PMIA and BT/PMIA electrospun fibers and their diameter distributions.

**Figure S7.** **Cross-sectional SEM images of (a)** 10FG and **(b)** 50FG composite papers. **(c)** Functional demonstration of 20FG composite paper. **(d)** FTIR spectra of different samples as indicated. **(e)** Peak fitting of pure PMIA and 50FG composite papers. **(f)** XRD patterns of pure PMIA and 50FG composite papers.

**Figures S7a,b** present the cross-sectional morphologies of the composite papers with varying FG contents. Comparing the fiber mats and the corresponding hot-pressed papers demonstrates that hot pressing substantially reduces the porosity of the fibrous network, yielding a highly densified structure. Specifically, the originally loose, porous fibrous architecture is converted to a smooth, continuous surface morphology, confirming that hot pressing effectively facilitates inter-fiber bonding and fusion. The internal fibers of the pure PMIA paper are mutually entangled, exhibiting a relatively uniform cross-sectional morphology without discernible defects, which accounts for its good continuity and flexibility. In the 10FG paper, the FG nanosheets exhibit a predominantly in-plane orientation and form strong interfacial bonding with the PMIA matrix. However, the drastically elevated filler contents and excessive incorporation of 2D nanosheets in the 50FG paper disrupt the continuity of the PMIA fiber network. This disruption gives rise to more prominent lamellar undulations and roughened regions in the cross-section, accompanied by local stacking, agglomeration, and increased porosity. These morphological observations collectively confirm that excessive filler contents induce structural discontinuity and the formation of localized defects.

Furthermore, as illustrated in **Figure S7c**, the 20FG composite papers exhibit exceptional mechanical flexibility, maintaining their structural integrity even under large-scale bending and folding.

**Figure S7d** presents the FTIR spectra of the pure PMIA, 10FG, and 50FG composite papers, and neat FG filler, within the range of 400–4000 cm⁻¹. Both the pure PMIA and the two composite papers exhibit the characteristic absorption bands of meta-aramid backbones.^[4, 5]^ Specifically, the region around 3285–3266 cm⁻¹ corresponds to the N–H stretching vibration. The peak at 1649 cm⁻¹ represents the Amide I band, which is primarily associated with the C=O...H stretching vibration. The band at 1604 cm⁻¹ is related to the C=C stretching vibration of the aromatic rings, while the peak at 1528 cm⁻¹ is identified as the Amide II band, corresponding to the N–H in-plane bending vibration. Notably, both the PMIA and FG-based papers exhibit distinct peak splitting within the Amide I and Amide II band regions.

The characteristic peak positions of the papers remain consistent overall, suggesting that the incorporation of FG does not alter the chemical structure of the PMIA molecular chains but primarily influences the local environment of the amide groups. Meanwhile, the N–H stretching peak shifts toward lower wavenumbers, from 3285 cm^-1^ for pure PMIA to 3269 cm^-1^ for the 10FG paper and 3266 cm^-1^ for the 50FG paper, exhibiting a clear redshift. This redshift is typically associated with the enhancement of hydrogen bonding and can be attributed to the interfacial interactions between FG filler and PMIA matrix, which restrict the vibration of the amino groups and thereby reduce their characteristic vibrational frequency.^[4, 6]^ The covalent C–F stretching vibration peak of FG appears near 1207 cm^-1^. However, this peak partially overlaps with the C–N stretching vibration peak of PMIA at approximately 1240 cm^-1^, making it difficult to resolve directly in the FTIR spectra.^[7]^ To confirm the fluorinated structural features of the composite papers, peak fitting was performed on the spectra of the PMIA and 50FG composite papers (**Figure S7e**). The results reveal a distinct characteristic peak at 1207 cm^-1^ for the 50FG composite paper, which is assigned to the C–F stretching vibration.^[8, 9]^

**Figure S7f** displays the XRD patterns of the PMIA and 50FG paper. For the pure PMIA paper, a prominent diffraction peak appears at *2θ*=22.8°, which is the characteristic peak of PMIA assigned to the (200) crystal plane of the crystalline structure.^[5, 10]^ In the 50FG composite paper, new diffraction peaks emerge at *2θ*=12.2° and 41.2°, corresponding to the (001) and (100) planes of FG, respectively. The (001) peak is associated with the interlayer ordering along the stacking direction, while the (100) peak characterizes the in-plane dimension of the C–C network.^[1, 11]^ The presence of these characteristic FG peaks in the composite paper substantiates the successful incorporation of FG into the PMIA matrix.

**Figure S8. (a)** Trap parameters and **(b)** normalized surface potential curves of pure PMIA and different FG/PMIA composite papers.

To eliminate the interference of inherent differences in initial surface potential across different samples and enable a quantitative comparison of surface potential decay rates, all measured raw data were normalized prior to analysis. The normalized surface potential *U*_N_(*t*) is defined as:

$$U_{N}\left(t\right)=\frac{U\left(t\right)}{U_{0}}$$

where *U*(*t*) is the actual surface potential, and *U*_0_ is the initial surface potential.

To quantitatively compare the differences in charge dissipation of composite papers with different filler contents from the perspective of carrier mobility, the carrier mobility of the papers can be calculated based on **Equation 2**:

$$\mu=\frac{L^{2}}{t_{r}U_{0}}$$

where *L* is the thickness of the paper, *U_0_* is the initial surface potential, and *t_r_* is the average time of carrier migration obtained through trap fitting. Here, *μ* represents the apparent mobility.

**Table S1. Trap density and peak trap energy level of PMIA and FG/PMIA composite papers**

| Sample | Shallow trap density | Deep trap density | Total trap density | Shallow trap energy  center (eV) | Deep trap energy  center (eV) |
| --- | --- | --- | --- | --- | --- |
| PMIA | 1.32×10^17^ | 4.60×10^16^ | 1.78×10^17^ | 0.9103 | 0.9646 |
| 10FG | 1.38×10^17^ | 7.15×10^16^ | 2.09×10^17^ | 0.9130 | 0.9729 |
| 20FG | 1.40×10^17^ | 8.63×10^16^ | 2.27×10^17^ | 0.9105 | 0.9783 |
| 35FG | 1.79×10^17^ | 6.18×10^16^ | 2.41×10^17^ | 0.8924 | 0.9565 |
| 50FG | 1.75×10^17^ | 4.03×10^16^ | 2.15×10^17^ | 0.8878 | 0.9529 |

For quantitative analysis, trap levels within 0.75–0.95 eV are defined as shallow traps, while those within 0.95–1.05 eV are defined as deep traps. The number of traps is obtained by integrating within the corresponding energy level ranges:^[12]^

$$n_{s}=\int_{0.75}^{0.95} N_{t}\left(E\right)dE$$

$$n_{d}=\int_{0.95}^{1.05} N_{t}\left(E\right)dE$$

where *n*_s_ is the number of shallow traps, and *n*_d_ is the number of deep traps.

**Table S2. Equivalent trap parameters of PMIA and FG/PMIA composite papers**

|  | *E*_trap_(eV) | *Q*_trap_(nC) |
| --- | --- | --- |
| PMIA | 0.75 | 6.28 |
| 20FG | 0.82 | 7.41 |
| 50FG | 0.74 | 12.73 |

**Figure S9. (a)** Failure probability of dielectric breakdown deduced from the Weibull distribution of the FG/PMIA composite papers. **(b)** Thermal conductivity and its enhancement of FG/PMIA composite papers. **(c)** The dielectric constant, **(d)** dielectric loss and **(e)** electrical modulus diagrams of the FG/PMIA composite papers.

**Figure S9a** illustrates the dielectric breakdown strength of the PMIA and various FG/PMIA composite papers. The breakdown strength of the pure PMIA paper is 116.27 kV/mm, and the breakdown characteristics of the FG papers exhibit a rising-then-falling trend with increasing FG content. Notably, the 20FG paper achieves a maximum value of 187.89 kV/mm, which is approximately 1.62 times that of the neat PMIA paper. **Figure S9b** presents the thermal conductivity and the corresponding enhancement rates for the PMIA and various FG/PMIA composite papers. Both the thermal conductivity and its enhancement rate exhibit a continuous upward trend with increasing FG content, with far more pronounced improvement at low filler content. The thermal conductivity of the pure PMIA paper is 4.58 W∙m^-1^∙K^-1^, which increases to 8.26 W∙m^-1^∙K^-1^ at 20 wt% FG content. At higher concentrations, the thermal conductivity continues to rise but with a diminished growth rate; the 50FG composite paper does not reach the 10 W∙m^-1^∙K^-1^ threshold, representing an approximate 1.15-fold enhancement compared to the neat PMIA.

**Figure S9c** illustrates the dielectric constant of the FG/PMIA composite papers within the frequency range of 10–10^6^ Hz. Compared with the pure PMIA paper, the FG/PMIA composite papers exhibit an overall lower dielectric constant. This is attributed to the low intrinsic dielectric constant of FG and the low polarizability of C–F bonds. Furthermore, the incorporation of FG restricts the segmental movement of PMIA chains, thereby reducing the polarization capability of the composite papers. Regarding the internal properties of the FG/PMIA composite papers, the dielectric constant first increases and then decreases with rising FG content. This non-monotonic trend results from the competitive interplay between FG-induced suppression of matrix polarization and the enhanced micro-capacitor effect introduced by fillers. Similarly, the dielectric loss of the FG papers follows a rising-then-falling trajectory (**Figure S9d**), reaching a maximum value at 35 wt% FG content. The amplitude of the dielectric response varies with FG content (**Figure S9e**). The 35FG paper exhibits the highest amplitude, while that of the 50FG paper is slightly lower. These variations indicate that the FG content significantly dictates the intensity of loss-associated dielectric behavior.

Overall, the incorporation of FG not only reduces the permittivity of the composite papers but also modulates their dielectric loss behavior by tuning interfacial polarization and the formation of micro-capacitor structures.

**Figure S10. SEM image of the cross-section of the (a)** pure PMIA, **(b)** 5BT and **(c)** 20BT composite papers. **(d)** FTIR spectra of BNNT, pure PMIA, 5BT, and 20BT composite papers. **(e)** FTIR spectra of the amide bands of 5BT paper, 20BT paper and PMIA paper. **(f)** XRD patterns of PMIA and 20BT papers.

**Figures S10a–c** display the cross-sectional SEM micrographs of the PMIA, 5BT, and 20BT composite papers after hot-pressing densification. In the PMIA paper, residual fibrous traces are observed on the cross-section. During cryogenic brittle fracture sample preparation, PMIA fibers undergo tearing and mutual adhesion, resulting in an irregular fracture morphology. With the introduction of BNNT, the 5BT composite paper exhibits a relatively uniform filler distribution within the matrix, with prominent tubular ends and fine filament-like structures observed on the fractured surface. These morphological features confirm the formation of favorable interfacial bonding between the BNNT and the PMIA matrix, as the fillers typically experience a sequence of interfacial debonding and pull-out before complete separation during the fracture process.

**Figure S10d** presents the FTIR spectra of the PMIA paper, different BT/PMIA composite papers, and BNNT. Similar to the FG-based papers, the typical characteristic absorption peaks of PMIA appear in all BT/PMIA composite papers, indicating that the main chain structure of the PMIA matrix remains unchanged.^[5]^ The BNNT exhibit distinct characteristic peaks at 1337 cm^-1^ and 775 cm^-1^. Specifically, the peak at 775 cm^-1^ corresponds to the *A*_2u_ mode, which is associated with B–N vibrations perpendicular to the tube axis, while the peak at 1337 cm^-1^ corresponds to the *E*_1u_ mode, related to B–N vibrations along the tube axis.^[8]^ In the composite papers, the appearance of absorption peaks near 1356 cm⁻¹ (for 5BT) and 1365 cm⁻¹ (for 20BT) corresponds to the characteristic features of BNNT, providing clear evidence for their incorporation. In contrast, the distinguishability of the absorption peak near 775 cm^-1^ is relatively low. This is attributed to the spectral interference from the out-of-plane C–H vibration of PMIA at approximately 780 cm^-1^, which overlaps with the A_2u_ characteristic peak of the BNNT, rendering the peak profile in this region difficult to resolve directly.^[7]^ Furthermore, compared with the PMIA paper, both the Amide I and Amide II bands of the BT/PMIA composite papers exhibit a shift toward lower wavenumbers (**Figure S10e**). Specifically, the Amide I band moves from 1649 cm^-1^ in the PMIA paper to 1645 cm^-1^ for the 5BT paper and 1647.8 cm^-1^ for the 20BT paper. Similarly, the Amide II band shifts from 1529 cm^-1^ in the PMIA paper to 1525.5 cm^-1^ for both the 5BT and 20BT papers. This redshift indicates that the introduction of BNNT has altered the local chemical environment of the PMIA amide bonds.^[13]^ Concurrently, the N–H stretching vibration peak shifts from 3285 cm^-1^ in the PMIA paper to 3270 cm^-1^ for the 5BT paper and 3278 cm^-1^ for the 20BT paper. The redshift of the N–H peak is typically associated with the enhancement of hydrogen bonding, suggesting a potential strengthening of the BNNT/PMIA interfacial interaction.^[9]^

To verify the incorporation of BNNT from a crystallographic perspective, XRD characterization was performed on the PMIA and 20BT papers, as illustrated in **Figure S10f**. Compared with the pure PMIA, the 20BT paper exhibits a prominent diffraction peak at 25.5°, which corresponds to the (002) crystal plane of the BNNT. Additionally, characteristic peaks at 40.6° and 53.7° are observed, assigned to the (100) and (004) planes, respectively. The emergence of these BNNT-specific diffraction peaks in the composite paper substantiates the successful introduction of BNNT into the PMIA matrix.

**Figure S11. (a)** Normalized surface potential curves and **(b)** trap distribution curves of PMIA and different BT/PMIA composite papers. **(c)** Trap density and peak trap energy level of PMIA and BT/PMIA composite papers.

For the different BT/PMIA composite papers, this study primarily utilizes ISPD and TSDC analyses to investigate the relationship between trap parameters and breakdown performance. **Figure S11a** displays the normalized surface potential decay (SPD) curves of the BT/PMIA papers. After normalization, the potential decay rates of the composite papers can be compared directly. The neat PMIA paper exhibits the most rapid potential decay. With increasing BNNT content, the decay rate of the BT/PMIA papers gradually slows down, maintaining higher normalized surface potentials throughout the 0~3200 s interval. Specifically, the 20BT paper possesses the highest normalized potential, while the decay curves for the 10BT and 15BT papers are relatively close. This result is attributed to the fact that the introduction of BNNT facilitates charge trapping and inhibits the de-trapping (release) process, thereby decelerating the potential decay. **Figure S11b** also displays the fitted trap distribution curves of the BT/PMIA papers. Compared with the neat PMIA paper, the deep trap peaks of the BT/PMIA papers shift toward higher energy levels, while the amplitude of the shallow trap peaks gradually diminishes. The statistical results of the trap parameters for the BT/PMIA papers are further summarized in **Table S3** and **Figure S11c**.

Compared with the pure PMIA paper, both the deep and shallow trap energy levels of the BT/PMIA papers exhibit an overall upward trend with increasing BNNT content. The quantity of deep traps increases overall, whereas the number of shallow traps continuously decreases, keeping the total trap density at a consistently high level. Generally, the BT/PMIA papers are dominated by deep traps in terms of both energy level and quantity.

**Table S3. Trap density and peak trap energy level of PMIA and BT/PMIA composite papers**

| Sample | Shallow trap density | Deep trap density | Total trap density | Shallow trap energy  center (eV) | Deep trap energy  center (eV) |
| --- | --- | --- | --- | --- | --- |
| PMIA | 1.32×10^17^ | 4.60×10^16^ | 1.78×10^17^ | 0.9103 | 0.9646 |
| 5BT | 1.09×10^17^ | 1.06×10^17^ | 2.15×10^17^ | 0.9209 | 0.9880 |
| 10BT | 9.02×10^16^ | 1.00×10^17^ | 1.91×10^17^ | 0.9314 | 0.9859 |
| 15BT | 8.53×10^16^ | 1.04×10^17^ | 1.90×10^17^ | 0.9290 | 0.9873 |
| 20BT | 7.41×10^16^ | 1.10×10^17^ | 1.84×10^17^ | 0.9304 | 0.9958 |

**Figure S12.** Simulated spatiotemporal evolution of electrical tree propagation and local electric field distributions for **(a)** pure PMIA and **(b)** 15BT composite papers under a continuously applied electric field.

To elucidate the dynamic evolution of electrical breakdown paths within the dielectrics, quasi-transient finite element analysis was performed based on the electrostatic formulation. The spatial electric field distribution at each discrete growth step was determined by solving Gauss’s law for electricity, utilizing the electric potential as the primary dependent variable. The governing electrostatic equations are defined as follows:

$$-\nabla\cdot\left(\varepsilon_{0}\varepsilon_{r}\nabla T\right)=\rho_{v}$$

$$\nabla\cdot D=\rho_{v}$$

$$D=\varepsilon_{0}\varepsilon_{r}E$$

$$E=-\nabla V$$

where *ε*_0_ is the vacuum dielectric constant, *ε*_r_ is the relative dielectric constant, *V* is the electric potential, *ρ*_v_ is the electric charge density, *D* is the potential shift field, *E* is the electric field. The simulation domain was constructed as a 2D grid scaled to 1200 × 600 (length × width). The model boundary conditions were defined by applying a constant high-voltage excitation to the bottom boundary, grounding the top boundary, and assigning a zero-charge boundary condition to both lateral sides. The intrinsic dielectric properties of the composite components were defined by assigning relative dielectric constants of 1.6 for PMIA and 3.9 for BNNT. In the framework of the fractal medium breakdown and percolation model, the evolution probability *p* of the electric tree channel was defined as follows:

$$p\left(i',j'-i,j\right)=\alpha+\beta-\gamma$$

where *ϕ_f_* is the threshold potential for electrical breakdown of the dielectric, *ϕ_i_*_,_*_j_* , *ϕ_i’_*_,_*_j’_* and *ϕ_i”_*_,_*_j”_* are the potentials at the discharge point, the possible point and the link point, respectively, *η* is the fractal dimension, and *γ* denotes the evolutionary energy dissipation at the propagating tip. This model successfully validates the physical mechanism of enhancing the insulation performance of the composite paper through the BNNT-induced “barrier effect” that retards the propagation of electrical trees.

**Figure S13.** Schematic diagram of the mechanism of electrothermal synergy.

**Figure S14. (a)** Cross-sectional SEM image of the FBF composite paper. **(b)**Thickness statistics of each layer in the FBF composite paper. **(c)** Stress-strain curves, **(d)** strain at break and tensile stress graphs of FBF composite paper and PMIA paper.

As illustrated in **Figure S14a**, the FBF composite paper adopts a symmetric sandwich structural design, with cross-sectional SEM morphology revealing tightly bonded interfaces between adjacent layers without discernible delamination or defects. To effectively analyze the structural parameters of the FBF composite paper, a statistical analysis of layer thicknesses was conducted using cross-sectional SEM images (**Figure S14b**). A total of 60 data points were collected across three independently prepared experimental batches. The average thicknesses of the top layer, core layer, and bottom layer were measured to be 23 μm, 50 μm, and 25 μm, respectively. The resulting total calculated thickness of 0.1 mm aligns closely with the experimental measurements obtained in the laboratory.

As shown in **Figure S14c**, compared with pure PMIA paper, the incorporation of FG and BNNT significantly improves both the tensile strength and elongation at break of the FBF composite paper. Its stress–strain curve exhibits a higher slope (indicating an elevated initial modulus) and a larger work-of-fracture. As statistically depicted in **Figure S14d**, pure PMIA paper exhibits a tensile strength of 20.95 MPa and a strain at break of 6.7%. In contrast, the tensile strength of the FBF composite paper increases substantially to 32.05 MPa, while maintaining a high strain at break of 6.9%. This is attributed to the hot-pressing temperature approaching the glass transition temperature (T_g_) of PMIA, which promotes macromolecular chain rearrangement and molecular-level fusion. Coupled with the uniform dispersion of nanofillers and interfacial hydrogen bonding, stress concentration is effectively suppressed.

**Table S4. Comparative analysis of the comprehensive performance of the FBF composite paper with other reported results.**

| Polymer matrix | Fillers | Fabrication strategy | λ  (W∙m^-1∙^K^-1^) | E_b_  (kV/mm) | Corona lifetime (min) | Tan δ | Operating temperature  (℃) | Ref. |
| --- | --- | --- | --- | --- | --- | --- | --- | --- |
| PMIA | FG  BNNT | Electrospinning and hot-pressing | 10.22 | 199.52 | 147.42 | < 0.05 | / | This work |
| Softwood paper | NFC | Three-layer hot-pressing | / | 186 | / | ~0.035 | 30-90 | [14] |
| Cellulose | CNC | Layer-by-layer spraying and hot-pressing | / | 108.97 | / | < 0.02 | / | [15] |
| PMIA | PET | Wet papermaking + sandwich assembly + hot-pressing | / | 56.35 | / | < 0.04 | / | [16] |
| PMIA | ANF | Vacuum filtration + hot-pressing | / | 82.7 | / | 0.06 | / | [17] |
| PMIA | PAR | Wet-laid papermaking and hot-pressing | / | 114.55 | / | < 0.04 | / | [18] |
| ANF | PEI  Mica | Spray-assisted gelation followed by pressure treatment | / | 176 | 26.5 | 0.03-0.06 | 250-300 | [19] |
| ANF | BNNS@PDA | Vacuum-assisted filtration and hot-pressing | 3.94 | / | / | / | / | [20] |
| ANF | ZNF | Vacuum-assisted filtration and hot-pressing (in situ growth) | / | 128 | / | 0.04 | / | [21] |
| ANF/AP | BNNS | Vacuum-assisted filtration and hot-pressing | 4.34 | 59.6 | / | < 0.02 | / | [22] |

To clarify the distinct architectural advantages of the FBF composite paper over previously reported sandwich-structured insulating composites, a comparative performance analysis was conducted based on the compiled data in **Table S4**. Traditional sandwich-structured insulating systems, such as softwood paper/NFC (NPN), cellulose/CNC, PMIA/PET, ANF/PMIA/ANF, and PAR/PMIA/PAR configurations, primarily focus on physical void filling, porosity reduction, or interfacial mechanical sealing through wet papermaking, vacuum filtration, or layer-by-layer spraying followed by standard hot-pressing. Consequently, these conventional systems omit thermal management entirely, resulting in low intrinsic thermal conductivity (λ) while remaining highly vulnerable to electric field concentration and polarization at the rough, non-chemically bonded interfaces. Although certain high-performance nanofiber composites like ANF/PEI/Mica exhibit improved breakdown strength (E_b_ = 176 kV/mm) and moderate thermal endurance, their resistance to surface partial discharge remains limited, with a corona lifetime of only 26.5 min. Furthermore, strategies relying on simple unzoned incorporation of thermal fillers, such as ANF/AP/BNNS, achieve moderate thermal conductivity (4.34 W∙m^-1∙^K^-1^) but suffer from severe dielectric degradation (E_b_ dropping sharply to 59.6 kV/mm) because randomly distributed conductive paths disrupt the matrix integrity and lower the barrier for electrical treeing.

In sharp contrast, the FBF composite paper prepared via integrated electrospinning and hot-pressing densification overcomes these fundamental “thermo-electrical” trade-offs through spatial functional decoupling. By localizing FG in the outer layers, high-density deep energy traps and physical barrier effects are introduced to suppress charge injection and surface partial discharge, extending the corona lifetime to 147.42 min. Concurrently, preferentially orienting BNNT exclusively within the core layer creates continuous, low-thermal-resistance phonon transport channels, yielding a superior thermal conductivity of 10.22 W∙m^-1∙^K^-1^ without impairing the surface electrical shielding. Crucially, the electrospinning process combined with thermal densification enables PMIA molecular chain rearrangement and molecular-level fusion across adjacent functional layers. This molecular fusion eliminates defective inter-layer boundaries, acoustic vibrational mismatch, and localized dielectric field concentration, maintaining an exceptionally high dielectric breakdown strength of 199.52 kV/mm and low dielectric loss (tan δ < 0.05). Overall, the hierarchical synergy of “surface barrier–core phonon pathway–interlayer molecular fusion” allows the FBF composite paper to simultaneously achieve elite thermal transport, high dielectric strength, and prolonged corona durability at low overall filler loadings, demonstrating clear technological superiority over conventional sandwich dielectric designs.

**Figure S15.** Three-dimensional morphology diagrams of **(a)** FBF composite paper, **(b)** Nomex 410 and **(c)** TH paper. **(d)** The surface arithmetical mean height (*Sa*) and maximum height (*Sz*) of FBF composite paper, Nomex 410 and TH paper.

**Figure S16. Schematic diagram of finite element simulation modeling of the temperature field of (a)** neat PMIA paper, **(b)** disordered mixture paper, **(c)** ordered mixture paper and **(d)** FBF sandwich paper.

Finite element simulation analysis was performed using COMSOL Multiphysics 6.2. The construction process of the four paper simulation models is illustrated in **Figure S13**. Rectangles were set as the PMIA matrix for the four papers, with a measured thermal conductivity λ of 4.58 W·m⁻¹·K⁻¹. Ellipses represent the FG filler with a λ of 50 W·m⁻¹·K⁻¹, and small rectangles represent the BNNT filler with a λ of 400 W·m⁻¹·K⁻¹.

The interfacial thermal resistance of the surface and core layers was derived by combining the Foygel model with Effective Medium Theory (EMT), where the formula for the EMT model is expressed as follows:

$$K_{\text{c}}=K_{p}\frac{\left[2\left(\frac{K_{f}}{K_{p}}-\frac{K_{f}}{ah_{c}}-1\right)V_{f}+\frac{K_{f}}{K_{p}}+\frac{2K_{f}}{ah_{c}}+2\right]}{\left[\left(1-\frac{K_{f}}{K_{p}}+\frac{K_{f}}{ah_{c}}\right)V_{f}+\frac{K_{f}}{K_{p}}+\frac{2K_{f}}{ah_{c}}+2\right]}$$

Where *K_c_*, *K_p_*, and *K_f_* represent the thermal conductivities of the composite, polymer matrix, and filler, respectively. *V_f_* is the volume fraction of the filler，*a* is the particle radius of the filler，and *h_c_* denotes the interfacial thermal conductance, whose reciprocal represents the interfacial thermal resistance(*R_B_*=1/*h_c_*). Based on the calculated results, the interfacial thermal resistances of FG/PMIA and the BT/PMIA layer were set to 1.86×10^-6^ m^2^·K·W^-1^ and 1.5×10^-8^ m^2^·K·W^-1^, respectively, within COMSOL.

A Monte Carlo algorithm was employed to randomly distribute these fillers, simulating their distribution characteristics within the PMIA matrix. The direct hybrid model was divided into disordered and ordered mixing types. The left surface of the simulation domain was set as a boundary heat source of 500 W/m², while the heat flux on the right surface originated from convective heat transfer induced by ambient cooling. The calculation formula is as follows:

$$n\cdot\lambda\nabla T=h\left(T_{ext}-T\right)$$

Except for the left and right surfaces, all other surfaces of the matrix were regarded as thermal insulators, and their boundary constraint equations are as follows:

$$n\cdot\lambda\nabla T=0$$

where *T* is the matrix temperature, *T*_ext_ is the ambient temperature, and $\text{h}$ is the convective heat transfer coefficient. The steady-state condition was calculated using the following equation:

$$\nabla\cdot\overrightarrow{q}=Q$$

$$\overrightarrow{q}=-\lambda\nabla T$$

where *q* is the heat flux and *Q* is the heat source.

**References**

1.	E. Pretsch, P. Bühlmann, and M. Badertscher, *Structure Determination of Organic Compounds*, 5th ed., Laboratory Manuals (Berlin: Springer, 2020), ISBN 9783662624388.

2.	J. M. García, F. C. García, F. Serna, and J. L. De La Peña, “High-Performance Aromatic Polyamides,” *Progress in Polymer Science* 35 (2010): 623–686. https://doi.org/10.1016/j.progpolymsci.2009.09.002.

3.	N. R. Babij, E. O. McCusker, G. T. Whiteker, et al., “NMR Chemical Shifts of Trace Impurities: Industrially Preferred Solvents Used in Process and Green Chemistry,” *Organic Process Research & Development* 20 (2016): 661–667. https://doi.org/10.1021/acs.oprd.5b00417.

4.	X. Du, Z. Chai, J. Zhang, et al., “Synergistic Enhancements of Mechanical and Insulating Properties in Electrospun PMIA-Based Composite Films,” *Journal of Physics D: Applied Physics* 58 (2025): 285501. https://doi.org/10.1088/1361-6463/ade9d5.

5.	Y. Li, J. Li, J. Liu, et al., “Ultra-Strong, Ultra-Tough, and Transparent Pure Poly (m-Phenylene Isophthalamide) (PMIA) Films Mediated by a Highly Ordered Aggregation Structure and Hydrogen-Bonded PMIA Crystal Structures,” *Chemical Engineering Journal* 515 (2025): 163601. https://doi.org/10.1016/j.cej.2025.163601.

6.	X. Wang and P. Wu, “Highly Thermally Conductive Fluorinated Graphene Films with Superior Electrical Insulation and Mechanical Flexibility,” *ACS Applied Materials & Interfaces* 11 (2019): 21946–21954. https://doi.org/10.1021/acsami.9b07377.

7.	X. Zhang, X. Tang, R. Wang, R. Wang, X. Yan, and M. Shi, “Thermal Degradation Behaviors and Fire Retardant Properties of Poly(1,3,4-Oxadiazole)s (POD) and Poly(m-Phenylene Isophthalamide) (PMIA) Fibers,” *Fibers and Polymers* 18 (2017): 1421–1430. https://doi.org/10.1007/s12221-017-1185-7.

8.	C. Zhang, R. Huang, P. Wang, et al., “Highly Compressible, Thermally Conductive, yet Electrically Insulating Fluorinated Graphene Aerogel,” *ACS Applied Materials & Interfaces* 12 (2020): 58170–58178. https://doi.org/10.1021/acsami.0c19628.

9.	N. A. T. Thieu, D. Mani, J. C. Won, K. Yon, M. C. Vu, and S. Kim, “Vertically Interconnected Network of Graphene Fluoride for Highly Thermoconductive and Electrically Insulating Epoxy Composites,” *Polymer Composites* 43 (2022): 8050–8059. https://doi.org/10.1002/pc.26946.

10.	K. Sun, F. Lv, W. Zhang, et al., “Self-Reinforced Doping Strategy in the Multiscale PMIA Paper for High Mechanical Properties and Insulating Performance,” *ACS Applied Materials & Interfaces* 15 (2023): 53902–53912. https://doi.org/10.1021/acsami.3c11566.

11.	Y. Chen, Z. Wang, K. Owusu-Sekyere, et al., “Morphology of Taylor Cone in Stable Regime: Experimental and Numerical Insights,” *Langmuir* 41 (2025): 24841–24856. https://doi.org/10.1021/acs.langmuir.5c03395.

12.	Q. Li, X. Li, Y. Yang, Y. Li, and G. Wu, “The Evolution of Trapping Parameters on Three-Layer Oil-Paper of Partial Discharge Degradation for on-Board Traction Transformers,” *IEEE Access* 8 (2020): 175651–175659. https://doi.org/10.1109/ACCESS.2020.3024774.

13.	N. S. Myshakina, Z. Ahmed, and S. A. Asher, “Dependence of Amide Vibrations on Hydrogen Bonding,” *The Journal of Physical Chemistry B* 112 (2008): 11873–11877. https://doi.org/10.1021/jp8057355.

14.	X. Huang, Y. Zhou, Q. Gesang, et al., “Construction of Nanocellulose Sandwich-Structured Insulating Paper and Its Enhancement for Mechanical and Electrical Properties,” *IEEE Transactions on Dielectrics and Electrical Insulation* 28 (2021): 1127–1135. https://doi.org/10.1109/TDEI.2021.009446.

15.	D. Zhang, S. Wang, J. Jiang, et al., “Enhancement of Mechanical and Electrical Properties of Multilayer Composite Nanocellulose-Modified Insulating Paper,” *IEEE Transactions on Dielectrics and Electrical Insulation* 33 (2026): 257–266. https://doi.org/10.1109/TDEI.2025.3645748.

16.	M. Chen, S. Yao, G. Zhu, et al., “High‐strength, Electrically Insulated Industrial Meta‐aramid Paper Reinforced with Polyethylene Terephthalate Microfibre Pulp in a Sandwich Structure,” *High Voltage* 9 (2024): 1402–1413. https://doi.org/10.1049/hve2.12474.

17.	G. Duan, F. Hu, Y. Liang, et al., “Novel Sandwich-Structured Flexible ANF/PMIA/ANF Composite Paper with Enhanced Breakdown Strength,” *Fibers and Polymers* 26 (2025): 1381–1391. https://doi.org/10.1007/s12221-025-00889-1.

18.	Y. Chen, H. Ma, J. Jiang, et al., “Hierarchical Sandwich‐structured Polyarylate Nanofiber/Meta‐aramid Nanocomposite with Robust Dielectric Strength, Mechanical Durability, and Environmental Stability,” *Polymer Composites* 47 (2026): 11880–11893. https://doi.org/10.1002/pc.71062.

19.	X. Pan, Z. Bao, W. Xu, et al., “Recyclable Nacre‐like Aramid‐mica Nanopapers with Enhanced Mechanical and Electrical Insulating Properties,” *Advanced Functional Materials* 33 (2023): 2210901. https://doi.org/10.1002/adfm.202210901.

20.	T. Ma, Y. Zhao, K. Ruan, et al., “Highly Thermal Conductivities, Excellent Mechanical Robustness and Flexibility, and Outstanding Thermal Stabilities of Aramid Nanofiber Composite Papers with Nacre-Mimetic Layered Structures,” *ACS Applied Materials & Interfaces* 12 (2020): 1677–1686. https://doi.org/10.1021/acsami.9b19844.

21.	B. Yang, B. Yuan, P. Xu, and M. Zhang, “Dual‐network Assembled Nanopaper towards Extremely Harsh Conditions,” *Advanced Functional Materials* 34 (2024): 2407763. https://doi.org/10.1002/adfm.202407763.

22.	M. Li, Y. Zhu, and C. Teng, “Facial Fabrication of Aramid Composite Insulating Paper with High Strength and Good Thermal Conductivity,” *Composites Communications* 21 (2020): 100370. https://doi.org/10.1016/j.coco.2020.100370.
